# Bioenergetics-based modeling of *Plasmodium falciparum* metabolism reveals its essential genes, nutritional requirements, and thermodynamic bottlenecks

**DOI:** 10.1371/journal.pcbi.1005397

**Published:** 2017-03-23

**Authors:** Anush Chiappino-Pepe, Stepan Tymoshenko, Meriç Ataman, Dominique Soldati-Favre, Vassily Hatzimanikatis

**Affiliations:** 1 Laboratory of Computational Systems Biotechnology, École Polytechnique Fédérale de Lausanne, EPFL, Lausanne, Switzerland; 2 Department of Microbiology and Molecular Medicine, Faculty of Medicine, University of Geneva, CMU, Geneva, Switzerland; The Pennsylvania State University, UNITED STATES

## Abstract

Novel antimalarial therapies are urgently needed for the fight against drug-resistant parasites. The metabolism of malaria parasites in infected cells is an attractive source of drug targets but is rather complex. Computational methods can handle this complexity and allow integrative analyses of cell metabolism. In this study, we present a genome-scale metabolic model (iPfa) of the deadliest malaria parasite, *Plasmodium falciparum*, and its thermodynamics-based flux analysis (TFA). Using previous absolute concentration data of the intraerythrocytic parasite, we applied TFA to iPfa and predicted up to 63 essential genes and 26 essential pairs of genes. Of the 63 genes, 35 have been experimentally validated and reported in the literature, and 28 have not been experimentally tested and include previously hypothesized or novel predictions of essential metabolic capabilities. Without metabolomics data, four of the genes would have been incorrectly predicted to be non-essential. TFA also indicated that substrate channeling should exist in two metabolic pathways to ensure the thermodynamic feasibility of the flux. Finally, analysis of the metabolic capabilities of *P*. *falciparum* led to the identification of both the minimal nutritional requirements and the genes that can become indispensable upon substrate inaccessibility. This model provides novel insight into the metabolic needs and capabilities of the malaria parasite and highlights metabolites and pathways that should be measured and characterized to identify potential thermodynamic bottlenecks and substrate channeling. The hypotheses presented seek to guide experimental studies to facilitate a better understanding of the parasite metabolism and the identification of targets for more efficient intervention.

## Author summary

Almost half of the world population is at risk of infection by malaria parasites. The rise in drug-resistant parasites requires better understanding and targeting of their metabolism. In this study, we present a genome-scale metabolic reconstruction (iPfa) of the deadliest malaria parasite, *Plasmodium falciparum*, and its thermodynamics-based flux analysis (TFA). Our results support and extend the available experimental evidence on the essential genes and nutritional requirements of this organism. Besides, we identify metabolites that give rise to thermodynamic bottlenecks and suggest substrate channeling. Overall, these results provide novel insight into the metabolism of *P*. *falciparum* and may guide experimental studies to develop a better characterization of the parasite metabolism and the identification of antimalarial drug targets.

## Introduction

Malaria remains a major global health care concern, with almost half of the world population at risk of infection that ultimately results in over half a million deaths each year [1]. Of the five *Plasmodium* species capable of infecting humans, *P*. *falciparum* is responsible for most malaria-related deaths. The current increase in parasites with resistance to most of the clinically used antimalarial drugs, including artemisinin, renders the treatment of this disease more challenging [1]. The development of more efficient antimalarial treatments is, therefore, a highly pressing need. Because it is essential for cell development, metabolism represents a potential source for identifying novel targets. Computational methods can handle its complexity and thus facilitate the discovery of drug targets (as demonstrated for other pathogens [2, 3]) that are particularly interesting for malaria research.

Genome-scale metabolic models (GEMs) represent an invaluable platform for the integrative analysis of cell metabolism [4]. Currently, two lineages of independently developed GEMs exist for *P*. *falciparum*, iTH366 [5] and PlasmoNet [6]. Since 2010, these GEMs have been slightly modified to study the metabolism of the parasite in the liver stage [7] or in the blood stages [8]. However, no study, to our knowledge, has developed an independent reconstruction of *P*. *falciparum* metabolism that updates, among other important features, the functional annotation of the genome, the localization of the enzymes and the definition of the available substrates according to the currently existing data.

The standard approach for analyzing different phenotypes using GEMs is flux balance analysis (FBA) [9, 10]. FBA predictions provide a good understanding of the metabolism at a systems level [11], and they can be further enhanced by integrating context-specific information in the form of constraints. FBA considers mass balance constraints for each metabolite in the metabolic network [9, 10]. Thermodynamics-based flux analysis (TFA) [12, 13] further accounts for thermodynamic constraints and provides a framework for the integration of metabolomics data in GEMs [12–17]. Thermodynamic constraints determine the feasible direction under which the reaction can operate, defined as reaction directionality [14–16].

Thermodynamic bottlenecks arise when alternative metabolic pathways are thermodynamically impeded although, based on network topology, they could have theoretically served a metabolic function. Unfavorable thermodynamics imposed by bulk-phase metabolite concentrations can be circumvented with substrate channeling. Substrate channeling involves the coupling of two or more reactions, and the common intermediate is transferred from the first enzyme to the second without escaping into the bulk phase. Such process has a major effect on the thermodynamics and kinetics of the involved catalytic functions and might determine specific responses to regulatory mechanisms. Techniques such as isotope dilution or enrichment, competing reaction or enzyme buffering have been traditionally used to detect and characterize substrate channeling for an enzyme pair or larger metabolon [18]. Such methods require systematic approaches, such as TFA, that generate and test thermodynamically consistent hypotheses to ultimately enhance our understanding of metabolism.

The primary goal of this study is to provide new insight into the essential metabolic capabilities and the nutritional requirements of *P*. *falciparum* that can reveal potential targets in its metabolism for efficient intervention. We also seek to identify metabolites whose intracellular concentrations give rise to thermodynamic bottlenecks and pathways where substrate channeling may exist. These analyses can guide metabolomics and biochemical studies on the metabolism of the parasite. For this purpose, we developed a GEM of *P*. *falciparum* (iPfa) and performed thermodynamically consistent studies using TFA and integrating the metabolite concentration ranges previously measured in intraerythrocytic *P*. *falciparum* [19–22]. We present here the TFA results for iPfa that suggest the essential genes, bottleneck metabolites, pathways with substrate channeling, and nutritional requirements of *P*. *falciparum*.

## Results

### Reconstruction of iPfa

We combined semi-automated approaches with a manual curation process based on the available literature on *P*. *falciparum* metabolism to reconstruct iPfa. A detailed description of the steps followed for the reconstruction process is provided in the S1 Methods. This process was performed in agreement with the standard protocol defined for the high-quality component-by-component (bottom-up) reconstruction of GEMs [23].

iPfa includes 325 genes and 670 metabolic reactions localized within five intracellular compartments: the cytosol, the mitochondrion, the apicoplast, the endoplasmic reticulum and the nucleus (Table 1). Nearly 13% of all metabolic reactions (excluding transport reactions) are orphans, i.e., enzymatic reactions that are not associated with any particular gene in the genome (S1 Methods). Orphan reactions in iPfa render feasible 24 of the 73 total metabolic tasks. Metabolic tasks are defined here as the production of biomass building blocks [3, 24]. The identified orphan reactions and alternatives might serve as a reference for further biochemical characterization of non-annotated genes in *Plasmodium* species (S1 Table).

**Table 1:** Description of iPfa.

**Metabolites**		**1258**	
Intracellular metabolites		1017	
	Cytoplasm		499
	Mitochondrion		171
	Apicoplast		209
	Endoplasmic reticulum		119
	Nucleus		19
Extracellular metabolites		241	
*Unique metabolites*		*673*	* *
**Reactions**		**1066**	
Metabolic reactions		670	
	Cytoplasm		374
	Mitochondrion		97
	Apicoplast		125
	Endoplasmic reticulum		67
	Nucleus		7
Transport reactions		396	
	Cytosol—extracellular		241
	Cytosol—mitochondrion		58
	Cytosol—apicoplast		58
	Cytosol—endoplasmic reticulum		34
	Cytosol—nucleus		5
*Unique metabolic reactions*		*558*	
**Gene-protein-reaction associations**		**586**	
	Cytoplasm		328
	Mitochondrion		83
	Apicoplast		113
	Endoplasmic reticulum		50
	Nucleus		7
	Transport		5
*Unique genes*		*325*	* *

**Fig 1:**
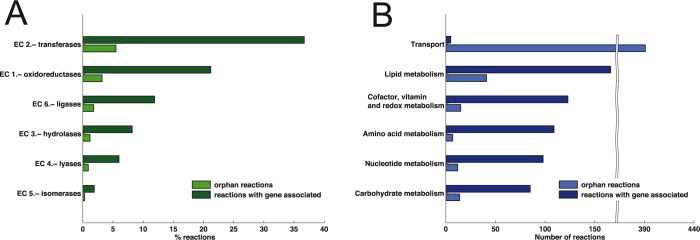
Metabolic capabilities (A) and subsystems (B) defined in iPfa.

iPfa also accounts for transport reactions: 236 potential uptakes, i.e. transports from the medium (host cell cytosol or blood serum) to the parasite’s cytosol, and 155 transports between intracellular compartments (Table 1). Due to the high uncertainty in the exact type and number of proteins that serve as transporters in *Plasmodium* spp., we included transport reactions based on a set of assumptions about the metabolite transportability (S1 Methods). These assumptions prevent the introduction of *ad hoc* constraints in iPfa and serve as an upper bound on the metabolite transportability. Such an approach leads to an underestimated number of essential genes. Nearly 98% of the transport reactions in iPfa remain orphan (Fig 1B).

The metabolic enzymes included in iPfa are distributed in six classes according to the E.C. identifiers. The majority of the metabolic enzymes in iPfa are transferases (43%), followed by oxidoreductases (24%), ligases (12%), hydrolases (12%), lyases (7%) and isomerases (2%) (Fig 1A). Regarding the metabolic subsystems, a significant percentage of metabolic reactions in iPfa can be classified as part of lipid metabolism (31%), followed by a broad and diverse group involving reactions related to the biosynthesis of cofactors, vitamins and redox molecules (21%). A similar number of metabolic reactions are involved in amino acid metabolism (17%), nucleotide metabolism (16%) and carbohydrate metabolism (15%) (Fig 1B).

The GEM iPfa presents an unbiased bottom-up reconstruction and an updated database of *P*. *falciparum* metabolism. For example, based on recent findings [25], the pyruvate dehydrogenase activity of the mitochondrial BCKDH complex was included in iPfa, whereas it was absent in the previous GEMs of the malaria parasite, iTH366 [5], PlasmoNet [6] and their modifications [7, 8] (see S1 Methods for more details). The coverage of metabolic enzymes (Fig 1A) is consistent with the ones reported in iTH366 and in the model of the related apicomplexan parasite *Toxoplasma gondii*, ToxoNet1 [3]. In contrast to the earlier GEMs of *P*. *falciparum*, iPfa is capable of growing on an *in silico* rich medium composed of 236 substrates that allow less restrictive conditions in the analyses. iPfa includes 245 common genes with iTH366 and 80 additional genes. Regarding metabolic tasks, iPfa accounts for 73 biomass building blocks of which 40 are common with iTH366 [5] and PlasmoNet [6], and nine are unique to iPfa. For example, some cofactors and the nucleotide sugars that are monomers of the essential GPI-anchor proteins [26] were not present in previous GEMs of *P*. *falciparum*. A detailed comparison of iPfa with the previous reconstructions iTH366 [5] and PlasmoNet [6] is provided in the S1 Methods.

We integrated various omics data in iPfa, which include available uptake and secretion rates of important metabolites, such as lactate [27], glucose [27] and L-isoleucine [28] (S1 Methods). Within the TFA framework, iPfa integrated the ranges of metabolite concentration measured at the blood stage of the malaria infection [19–22] to study the effect of the concentration ranges on the reaction directionality and gene essentiality. The results of these analyses validate that iPfa serves as a scaffold for the integration of context-specific information.

### Gene and reaction essentiality predictions per metabolic task with iPfa

We used iPfa to study single and double gene essentiality *in silico* (S2 Table and S3 Table). To understand the effect of the simulated knockouts on specific metabolic tasks of *P*. *falciparum*, we designed an analysis that identifies which metabolic tasks cannot be fulfilled in iPfa upon disruption of each *in silico* gene (Materials and Methods).

We first performed essentiality studies without accounting for thermodynamic constraints and identified 55 genes that are essential for growth (S2 Table). The genes are involved in 26 of the 73 total metabolic tasks that are defined in iPfa. The essentiality of 31 of the 55 genes has been previously validated experimentally through gene knockouts, suppressed transcription or inhibition of enzyme activity by drugs in *P*. *falciparum*, whereas the remaining 24 genes have not been examined empirically, to our knowledge. We grouped the 24 non-validated genes in two classes: the first class is composed of 14 genes that participate in metabolic pathways where other genes have been experimentally defined as essential, and the second class contains 10 genes that are associated with functions or pathways where other genes have not been reported as indispensable. Therefore, we could not hypothesize the latter class of genes to be essential based on previous context-dependent information. For example, the MEP/DOXP pathway in the apicoplast is essential in *P*. *falciparum* for the production of isopentenyl diphosphate and isoprenoid derivatives [29]. There are nine genes in iPfa that are involved in the MEP/DOXP pathway, and all nine genes are predicted as essential for the synthesis of three biomass building blocks: isopentenyl diphosphate, geranylgeranyl diphosphate and ubiquinone-8 (S2 Table). Experimental evidence validates the predicted essentiality of four of the nine genes [30–33], while the other five genes of the pathway remain to be tested and, based on our classification of non-validated genes, are allocated to the first class. The results of such studies could be used for further model validation and refinement. The second class involves genes that are essential for the synthesis of cardiolipin, ubiquinone-8 (downstream enzymes from the MEP/DOXP pathway), sugar nucleotides and nucleotides (S2 Table).

Next, we performed essentiality analysis using TFA, which includes information about the Gibbs free energy of reaction (Δ_r_G’) and allows the integration of metabolomics data (Materials and Methods). We applied two different approaches within the TFA framework. In the first approach, we allowed the concentration of every intracellular metabolite to vary between 1 μM and 50 mM. Besides, the concentration of extracellular metabolites (e.g., present in the parasitophorous vacuole, red blood cell or blood serum) varied between 0.01 μM and 100 mM. Such concentration limits are similar to the physiological ranges used in previous TFA studies [12, 13]. The constraints in the first TFA approach set 57 reactions in iPfa to be unidirectional (S4 Table), which allowed the identification of three additional essential genes (S2 Table). The three genes are involved in 12 tasks, including two tasks not identified with FBA, i.e., the production of GTP and phosphatidylserine. The essentiality of one of the genes, the gene encoding a serine palmitoyl transferase (E.C. 2.3.1.50), has been validated experimentally [34]. The other two genes encode the ATP:UMP and ATP:GMP phosphotransferases (E.C. 2.7.4.4 and E.C. 2.7.4.8), which were predicted to be essential for the tasks involving nucleotides and sugar nucleotides.

The study of the tasks using TFA provided additional insight into the effect of simulated knockouts on the growth. Earlier studies on the pyrimidine biosynthetic pathway have targeted the gene encoding the orotidine 5'-monophosphate decarboxylase (E.C. 4.1.1.23) with antimalarial inhibitors [35]. Despite the suggestions that the gene is only involved in the synthesis of the pyrimidine nucleotides CTP and UTP [35], TFA proposed an additional explanation for its essentiality: the disrupted synthesis of dCTP, dTTP, and the nucleotide sugars UDP-N-acetyl-D-glucosamine, UDP-glucose, and UDP-D-galactose (S2 Table). The identification of a high number of biomass building blocks impacted upon knockout of a gene suggests that the inhibitory effect of the antimalarial drug on the parasite’s growth is higher than believed. This result indicates a thermodynamic dependency between the production of phosphorylated and sugar nucleotides. The actual drug action mechanism should be further investigated with experiments and kinetic analysis.

In the second approach within the TFA framework, we integrated ten available metabolomics data sets, i.e., absolute concentrations measured in mature *P*. *falciparum* trophozoites [19–22] (Materials and Methods and S1 Methods). After the metabolomics data had been simultaneously integrated, 31 additional reactions in iPfa became unidirectional, and five additional genes were then identified to be essential (S1 Methods, S2 Table, and S4 Table). Interestingly, four of the genes had been assessed experimentally; three were found to be essential, and one was dispensable but growth reducing during the blood stages. The four genes encode a phosphatidyl serine carboxylase (E.C. 4.1.1.65) [36], a thioredoxin and glutathione oxidoreductase (E.C. 1.8.1.7) [37, 38] and a glycerol kinase (E.C. 2.7.1.30) [39]. The fifth gene, whose essentiality has not been assessed, encodes a glycerol 3-phosphate oxidoreductase (E.C. 1.1.1.21). All genes are associated with the production of phospholipids in the endoplasmic reticulum (ER) and are required to maintain the energy and redox balance in this compartment (see the next result section for further discussion). Although FBA identified alternative metabolic routes in iPfa for the production of phospholipids, TFA suggested that the alternative pathways were not thermodynamically feasible and that the five genes were essential. We also performed TFA with the simultaneous integration of the concentration data sets and identified 26 synthetic lethal pairs (S3 Table). Overall, the accuracy score of the gene essentiality predictions with iPfa (as defined in the Materials and Methods section) is 59%.

### TFA identifies bottleneck metabolites and suggests substrate channeling in iPfa

The number of reactions whose directionality was affected by thermodynamic constraints and the number of essential genes identified by TFA varied depending on the metabolite concentration ranges, and on the metabolomics data set used. With the generic concentration range, TFA predicted three additional essential genes compared with the FBA prediction. When each data set was independently integrated, three to five additional essential genes were identified (S2 Table). We evaluated the impact of the metabolite concentrations on the identification of the eight essential genes using TFA, and we identified *bottleneck metabolites* in iPfa. The *bottleneck metabolites* are the metabolites whose concentrations give rise to thermodynamic bottlenecks and are responsible for constraining the directionality of sets of reactions and for rendering any of these eight genes essential.

We first studied the effect of the generic concentration bounds on the prediction of the three essential genes. The lower and upper bound of the generic concentration range (1 μM—50 mM) were allowed to vary from 0.1 fM to 500 mM (Materials and Methods). While increasing the upper limit of the concentration range did not impact the essentiality of the three genes, decreasing the lower bound was critical for their essential function (Table 2).

**Table 2:** Bottleneck metabolites and affected reactions that determine the essential function of eight genes in iPfa.

Essential Gene (E.C.)	Min. set of bottleneck metabolites	Reaction impacted after integration of the bottleneck metabolites concentration ranges^1^ (Reaction directionalities^2^)	All bottleneck metabolites among alternatives	Data set description
*PF3D7_1415700* (2.3.1.50)	(i) CTP[r], Ethanolamine[c]	(Table SIII A and Table SIII D in S1 Methods)	CTP[r], Ethanolamine[c], Ethanolamine[r]	Required lower bound of 0.1 μM
	(i) CTP[r], Ethanolamine[r]			
*PF3D7_0111500* (2.7.4.-)	(i) UDP-N-acetyl-D-glucosamine[c], UTP[c], UDP-glucose[c], UDP-D-galactose[c]	(Table SIII B and Table SIII D in S1 Methods)	UDP-N-acetyl-D-glucosamine[c], UTP[c], UDP-glucose[c], UDP-D-galactose[c]	Required lower bound of 1 nM
*PF3D7_0928900* (2.7.4.8)	(i) GTP[c]	(Table SIII C and Table SIII D in S1 Methods)	Diphosphate[c], GTP[c], 2,5-Diaminopyrimidine nucleoside triphosphate[c], GDP-mannose[c]	Required lower bound of 1 fM
	(ii) 2,5-Diaminopyrimidine nucleoside triphosphate[c]			
*PF3D7_0927900* (4.1.1.65)	(i) CDP[r], CDP-ethanolamine[r], Ethanolamine phosphate[r]	R02057_r (B)	CDP[r], CDP-ethanolamine[r], Ethanolamine phosphate[r]	MS 2012 [21]
		R01468_r (B)		
		R02038_r (B)		
		T_c_to_r_C00189 (B)		
		R02055_r (F)		
		T_c_to_r_C00065 (F)		
		T_c_to_r_C00011 (R)		
*PF3D7_1351600* (2.7.1.30)	(i) CMP[r], Choline[r], AMP[r], ADP[r]	R01021_r (F)	CMP[r], Choline[r], AMP[r], CTP[r], ADP[r], ATP[r]	MS 2012 [21], NMR 2009 [19], NMR 2014 only strains 3D7, 7G8, C2(GC03), C4(Dd2), C6(7G8) [20]
		R01890_r (F)		
		R01321_r (F)		
	(ii) CMP[r], Choline[r], AMP[r], ATP[r]	R01280_r (F)		
		R00127_r (F)		
		R00094_r (F)		
	(iii) CMP[r], Choline[r], AMP[r], CTP[r]	T_c_to_e_C00116 (F)		
		T_c_to_r_C00111 (F)		
		R00842_r (R)		
	(iv) CMP[r], Choline[r], CTP[r], ADP[r]	R00847_r (R)		
		T_c_to_r_C00013 (R)		
		T_c_to_r_C00116 (R)		
*PF3D7_1419800*.*1* (1.8.1.7)	(i) AMP[r], ATP[r]	R00094_r (F)	ADP[r], AMP[r], CDP-choline[c], ATP[r], CTP[r], CTP[c], CMP[r]	All the data [19–21]
		R01280_r (F)		
*PF3D7_0923800*.*2* (1.8.1.7)		R00127_r (F)		
		T_c_to_r_C00111 (F)		
*PF3D7_1216200* (1.1.1.21)		R00842_r (R)		
		T_c_to_r_C00013 (R)		

^1^Reaction names as defined in iPfa. Metabolic reactions are defined with their R-5 digit identifier as obtained from the KEGG database. Transport reactions are marked with T_. Cellular compartments are defined with the reaction name: _r, endoplasmic reticulum; _c, cytosol; _e, extracellular (outside the parasite's cell).

^2^Reaction directionalities obtained with Thermodynamic Variability Analysis (TVA): (B) blocked, (F) forwards, (R) reverse. See the S4 Table for the reaction description.

When the intracellular concentrations were decreased and allowed to vary between 0.01 μM and 50 mM, the gene encoding a serine palmitoyl transferase (E.C. 2.3.1.50) was incorrectly predicted as non-essential [34]. The essential function of the ATP:UMP and ATP:GMP phosphotransferases (E.C. 2.7.4.4 and E.C. 2.7.4.8) was only lost when the lower bound of the intracellular concentrations was reduced four and ten orders of magnitudes, respectively, with reference to 1 μM. The available kinetic data, such as the Michaelis constant *K*_M_, provides a measure of the substrate concentration required for effective catalysis to occur. The *K*_M_ of the enzymes usually varies between 10^−1^ and 10^−7^ M. The *K*_M_ values found in the literature indicate that metabolite concentrations of 1 fM are not physiologically relevant, which further supports the essential function of the *P*. *falciparum* genes predicted with TFA and generic concentration ranges.

We next sought the bottleneck metabolites and the associated reactions in iPfa whose directionalities were constrained and led to the essential function of the genes (Materials and Methods). The analysis of bottleneck metabolites suggests that AMP and ATP constitute a single minimal set of metabolites that is responsible for rendering three genes essential (Table 2). The three genes associated with the bottleneck metabolites AMP and ATP are essential for maintaining the AMP/ATP ratio in the ER, which varies between 2.4 and 1.9 mol/L_cell_ / mol/L_cell_ based on the absolute minimum and maximum experimentally measured concentrations [19–22] (S1 Methods).

We also found four alternative minimal sets of metabolites that are responsible for the essential function of one gene of the five genes that become essential with TFA and metabolomics data. Each of these four sets includes four metabolites from an overall set containing CMP, choline, AMP, ATP, ADP and CTP. The bottleneck analysis allowed the identification of metabolites involved in additional alternative sets of larger size (Materials and Methods, Table 2).

Overall, we identified 18 bottleneck metabolites whose concentration ranges played a critical role in the directionality of reactions in the ER and cytosol (Table 2). The bottleneck metabolites are phosphorylated nucleotides, sugar nucleotides, and intermediates in the production of phospholipids that link the pyrimidine, aminosugar and phospholipid metabolism in *P*. *falciparum*.

**Fig 2:**
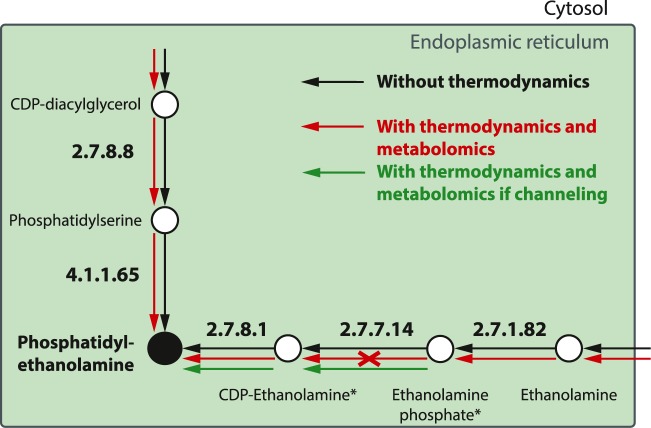
Bottleneck metabolites and substrate channeling in the production of PE. Arrows denote reaction fluxes. Circles define metabolites. Reactions are described by their 4-digit E.C. identifier. (*) are bottleneck metabolites.

Two bottleneck metabolites are CTP and ethanolamine, which are involved in the synthesis of phosphatidylethanolamine (PE) from ethanolamine through the Kennedy pathway (Fig 2). The bottleneck analysis indicates that the directionality of the reactions and the essential function of the gene products in the ER and the Kennedy pathway are very sensitive to the concentration values of CTP and ethanolamine. These metabolites impact the metabolism with generic (broad) and experimentally measured (narrow) concentration values (Table 2).

TFA with metabolomics data rendered the Kennedy pathway thermodynamically infeasible. However, the genes of *P*. *falciparum* involved in the Kennedy pathway are expressed in the blood stages [40], and evidence reported in the literature indicates that the Kennedy pathway is essential in the rodent malaria parasite *Plasmodium berghei* [41], which further suggests that the pathway is functional. The analysis of thermodynamic bottlenecks identified CDP-ethanolamine, CDP and ethanolamine phosphate as the bottleneck metabolites that block the reaction from CDP-ethanolamine to PE in the Kennedy pathway. Previous studies have also identified a thermodynamic impediment in the Kennedy pathway based on metabolite concentrations and have suggested channeling between ethanolamine phosphate and PE through the enzymes E.C. 2.7.7.14 and E.C. 2.7.8.1 [42]. When we simulated the presence of such channeling in TFA (Materials and Methods and S1 Methods), the Kennedy pathway became feasible (Fig 2) and the phosphatidylserine carboxylase (E.C. 4.1.1.65) is predicted as non-essential (further discussion in S1 Methods). These results demonstrate how TFA and the bottleneck metabolite analysis can be used to integrate biochemical studies and hypothesis testing.

### Study of nutritional requirements with iPfa

Obligate intracellular parasites, such as *P*. *falciparum*, depend on the availability of a broad array of metabolites provided by the host cell and its surroundings. To characterize the nutritional requirements of *P*. *falciparum*, we followed and extended the approach previously applied to the related pathogen *T*. *gondii* to study the *in silico minimal medium* (IMM) [3].

#### The *in silico* minimal medium (IMM) in iPfa

We used iPfa and an *in silico* rich medium composed of 236 substrates (S1 Methods) and searched for the IMM, defined as the minimum number of substrates required for growth [3]. The minimum number of substrates was 23, and there are 10,032 such IMM sets, which are generated by the alternative combination of only 52 substrates (S1 Methods, Table 3). We further identified 16 constitutive substrates, i.e., metabolites that were present in all alternative IMMs, and 36 non-constitutive substrates, i.e., metabolites that varied in each alternative IMM. In theory, one would expect 36!/((36–7)!*7!) = 8,347,680 combinations (S1 Methods); the relatively small number of alternatives suggests a limited ability of the 36 substrates to substitute for each other.

**Table 3:** Composition of the *in silico* minimal media (IMMs) that allow growth of iPfa and essentiality.

**Constitutive metabolites of the IMM**		Essential^1^
Source of amino acids	L-Isoleucine; Oxyhemoglobin	E; NE
Source of inorganic sulfur	Sulfate; Sulfur donor	E; E
Source of inorganic iron	Heme	NE^2^
Cofactors and others not synthesized *de novo*	Biotin; Methylcobalamin; Thiamine; Cholesterol	E; E; E; E
Precursors for lipoylation	Lipoate; Apoprotein	E; E
Precursors of lipid components	Choline; N-Acylsphingosine	E; NE^2^
Precursor of FMN/FAD	Riboflavin	E
Precursors of isoprenoids	4-Hydroxybenzoate; HCO^3-^	E; E
**Non-constitutive metabolites of the IMM**^**3**^		
Source of inorganic phosphate	Orthophosphate or Diphosphate	NE^2^
Precursors of CoA	Pantothenate or Pantetheine or N-((R)-Pantothenoyl)-L-cysteine	E
Source of pyridine ring (* and source of carbon)	Nicotinate D-ribonucleoside* or N-Ribosylnicotinamide* or Nicotinate or Nicotinamide	E
Source of DNA nucleotides (* and source of carbon)	S-adenosyl-L-methionine* or S-adenosyl-L-homocysteine* or S-adenosylmethioninamine* or Se-adenosyl-L-selenohomocysteine* or Adenosine* or Inosine* or Guanosine* or Xanthosine* or Adenine or Guanine or Hypoxanthine or Xanthine	NE^2^
Source of folate and derivatives	Tetrahydrofolate (THF) or 5,10-MethyleneTHF or 10-FormylTHF or 5-MethylTHF or 5,10-MethenylTHF or Dihydrofolate or Folate or Dihydropteroate or 4-Amino-4-deoxychorismate or Chorismate or 4-Aminobenzoate	E
Source of pyrimidine ring (precursors of UMP)	Orotate or (S)-Dihydroorotate	E
Source of C2/C4 for acetyl-CoA (precursors of nucleotide sugars)	Acetate or L-2-Amino-3-oxobutanoic acid	E

^1^Phenotype observed in the simulation after one-by-one depletion of the substrate or group of substrates in the *in silico* rich medium of 236 substrates: E, essential; NE, non-essential.

^2^See the next result section for explanation.

^3^The 10,032 alternative IMMs are generated through the combination of the constitutive metabolites and one non-constitutive metabolite from each group reported in Table 3. Note that the following constraints in the combinations should be considered: (a) the IMM should provide a ribose-containing molecule, which serves as source of carbon (marked with * in Table 3) and (b) the presence of orthophosphate and S-adenosylmethioninamine in the same IMM is not allowed (S1 Methods for more details).

Next, we investigated the reasons for the limited number of alternatives. Given the requirement of minimal utilization of substrates in the IMM studies, one would expect that some of the non-constitutive substrates that do not appear in the same IMM could substitute for each other. We created groups of non-constitutive substrates that did not belong to the same IMM and found that 20 of the 36 components were substitutable, suggesting that each of these components serve a specific biosynthetic requirement. However, ten can be substituted by one or more than one substrate in the IMM, which indicates that they serve multiple biosynthetic needs. For example, S-adenosyl-L-methionine can be substituted by S-adenosyl-L-homocysteine alone, or by two substrates simultaneously. In the latter case, one substrate is a source of the purines, such as adenine, guanine, hypoxanthine or xanthine, and the other serves as a source of carbon in the form of a ribose, such as nicotinate D-ribonucleoside or N-ribosylnicotinamide (Materials and Methods). This result demonstrated that the non-appearance of two substrates in the same IMM is a necessary but not sufficient condition to define the substrate substitutability.

We further searched for a measure that could determine whether two substrates are substitutable and found that the molecular structure of the 36 non-constitutive substrates could provide information on this criterion. Specifically, we identified eight common molecular substructures (here referred as *backbone moieties*) among the non-constitutive substrates, which allowed us to cluster the 36 non-constitutive substrates of the IMM (Table 3). Twenty-six substrates contain a unique backbone moiety out of five backbone moieties (Table 3). Importantly, substrates that contain the same unique backbone moiety are substitutable. Ten substrates contain two out of three backbone moieties (Table 3). These substrates can be substituted by one or more than one substrate in the IMM, as in the case of S-adenosyl-L-methionine discussed above.

The identification of the backbone moieties sheds light on the metabolic capabilities of *P*. *falciparum*. Analysis of the metabolic fate of the backbone moieties suggested that the cell may not be able to synthesize these backbone moieties *de novo* and must scavenge them from the substrates provided by the host cell. For example, the IMM analysis correctly identified the following nutritional requirements: the substrates pantothenate [43], hypoxanthine [44] or nicotinate [45], which are provided by default *in vitro* to maintain the cultures of *P*. *falciparum* [46]. However, our analysis suggested that each of these molecules could be substituted with other derivatives (Table 3). Nicotinate belongs to a group of substitutable substrates, which includes nicotinamide, nicotinate D-ribonucleoside, and N-ribosylnicotinamide. The four substrates contain the pyridine ring as a backbone moiety and this ring is also preserved in the final NAD^+^ molecule and its derivatives. Similar observations were obtained for the remaining backbone moieties (see next result section). Additionally, intraerythrocytic parasites rely on the uptake of exogenous niacin to synthesize NAD^+^ via the canonical Preiss-Handler salvage pathway [45]; however, to our knowledge, no one has studied the possibility of its substitution with D-ribonucleoside or N-ribosylnicotinamide, or with other molecules that provide the corresponding backbone moiety required for growth. The malaria parasites might be able to use a medium with fewer metabolites that contain the essential backbone moieties. Hence, the knowledge of nutritional requirements based on molecular structures will be used to simplify to its maximum extent the medium formulation, design synthetic media [47], and study the uptake mechanism and the metabolic fate of the backbone moieties.

#### Essentiality of IMM components

Although the components of the IMM serve all the essential functions in iPfa, they may not be essential when a rich medium of 236 substrates is available because other substrates could substitute for them. We removed one constitutive substrate at a time from the rich medium and found that 13 of the 16 constitutive substrates were essential, i.e., they are also required in a rich medium (Table 3). The other three substrates are heme, oxyhemoglobin, and N-acylsphingosine. They need more than one alternative substrate for their synthesis by the cell, e.g., heme. Or they serve as a single source of multiple components that are available in the rich medium or can be derived from it, e.g., hemoglobin, a source of various amino acids that exist in the rich medium.

To identify the essential functions served by the non-constitutive components of the IMM (Table 3) in iPfa, we analyzed the essentiality of the backbone moieties present in these components. Here, we removed the whole set of molecules that contained a backbone moiety (one moiety at a time) from the rich medium and found that five of the eight backbone moieties identified in the IMM were essential. This result suggests two fulfilled conditions for the five backbone moieties. There is a lack of other molecules (besides the 36 non-constitutive substrates of the IMM, Table 3) in the rich medium that can directly provide the five moieties, and *P*. *falciparum* is unable to synthesize the moieties from any other substrate available in the rich medium.

**Fig 3:**
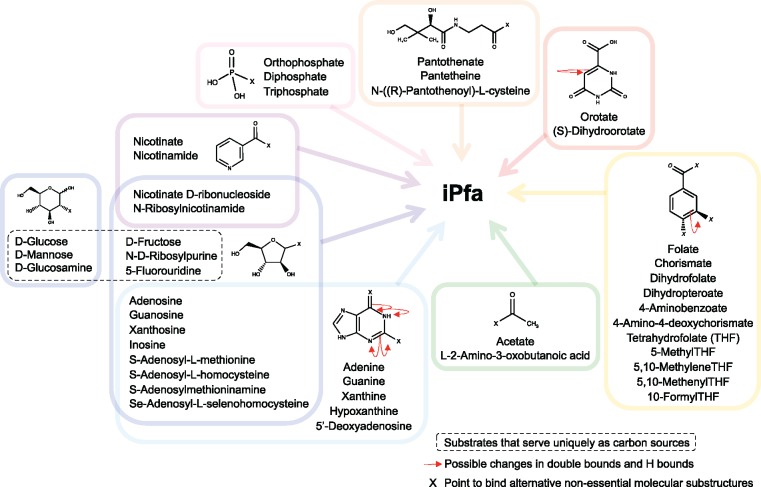
Nutritional requirements of *P*. *falciparum* concerning essential backbone moieties. Note: the presence of orthophosphate and S-adenosylmethioninamine in the same medium is not allowed. When *P*. *falciparum* grows on orthophosphate and S-adenosylmethioninamine as the only sources of phosphate and purines, it cannot synthesize enough ATP. The ATP limitation impedes the production of other phosphorylated nucleotides, sugar nucleotides, sphingomyelin and phospholipids in the stoichiometrically required amounts. As suggested throughout the manuscript, these metabolic processes are thermodynamically dependent (more details in the S1 Methods).

The essentiality analysis of the backbone moieties also indicates that other molecules (besides the 36 non-constitutive substrates of the IMM, Table 3) in the rich medium could provide three of the eight backbone moieties. The three backbone moieties are the sources of carbon, phosphate, and purines. We next performed iterative IMM analyses until we found new sets of substrates that provided the three backbone moieties (Fig 3). Interestingly, all of the new sets of substrates contained the three backbone moieties (Fig 3). The essentiality analysis (Materials and Methods) of all molecules that contained the three backbone moieties confirmed that these three backbone moieties are also essential. This observation suggests that iPfa does not possess the biosynthetic capabilities to produce the three backbone moieties and indicates that *P*. *falciparum* must scavenge the eight backbone moieties from the host cell or its surrounding.

We validated our results with available experimental work on the nutritional requirements of *P*. *falciparum* [43, 46, 48–51]. Although glucose, hypoxanthine, and pantothenate are known to be the preferred carbon, purine, and CoA sources respectively [43, 51], we have identified for the first time all alternative metabolites that are thermodynamically allowed to serve these functions (Fig 3). We further suggest that *P*. *falciparum* might use acetate or 2-amino-3-oxobutanoate to produce acetyl-CoA in the cytosol, which is then converted into the biomass building block UDP-N-acetyl-D-glucosamine.

Orotate is an intermediate metabolite in the canonical and highly conserved biosynthetic pathway of UMP, a common precursor of pyrimidine nucleotides. TFA of iPfa suggested that the biosynthetic pathway of pyrimidines is thermodynamically infeasible and hence the orotate backbone moiety, provided by orotate and (S)-dihydroorotate, needs to be taken up. However, the biosynthetic pathway of UMP, which involves six enzymes, is known to be functional [40] and essential in *Plasmodium* spp. given that the malaria parasites cannot salvage pyrimidines [50, 52]. We performed a comparative FBA against TFA to identify the thermodynamically constrained reaction(s) and found that the dihydroorotase reaction (DHOase, E.C. 3.5.2.3), which produces (S)-dihydroorotate from carbamoyl-L-aspartate, was not thermodynamically feasible. The malarial enzyme DHOase shares some characteristics with both types I (e.g., mammals) and II (e.g., *Escherichia coli*) enzymes [53]. Previous experimental [54] and computational [55] studies in DHOases of type II have hypothesized that channeling exists from carbamoyl phosphate to (S)-dihydroorotate through the enzymes E.C. 2.1.3.2 and E.C. 3.5.2.3. When TFA was used to simulate the presence of such channel in iPfa (Materials and Methods and S1 Methods), the pyrimidine biosynthetic pathway became thermodynamically feasible, and this backbone moiety could be synthesized by the parasite. Interestingly, previous attempts to develop the malaria parasites axenically have suggested that orotate incorporation can be used as a direct measurement of parasite development [50]. The presence of orotate in the medium has been shown to inhibit the function of the DHOase enzyme [53].

#### Gene essentiality studies in the IMM

The essentiality of the medium components suggests a complex interplay between the chemical structure of the substrates and the catalytic capabilities of the cell. To identify these links in *P*. *falciparum*, we evaluated single gene essentiality in iPfa for each of the alternative IMMs (S1 Methods). We identified 96 essential genes; the 63 genes predicted to be essential in the *in silico* rich medium (less restrictive condition) (S2 Table) remained indispensable in the alternative IMMs (more restrictive condition). We identified nine essential genes that were common in all IMMs (S5 Table), and the remaining 24 essential genes were distributed among the alternative IMMs with the number of genes varying among the alternative IMMs (S5 Table).

## Discussion

In this work, we reconstructed a GEM for *P*. *falciparum* and performed thermodynamics-based flux analysis to study the essential metabolic capabilities and the nutritional requirements of the parasite. Besides, we identified thermodynamic bottlenecks and hypothesized substrate channeling in the metabolic pathways. The results from this work, which have been compared with available experimental evidence, demonstrate that iPfa and its TFA can be used to fill in the knowledge gaps regarding the critical aspects of *P*. *falciparum* metabolism.

The highest accuracy score (59%) in the prediction of essential genes for growth was obtained using TFA with metabolomics data. Such approach revealed up to 63 lethal genes and 26 synthetic lethal pairs in iPfa (S2 Table and S3 Table). Existing empirical evidence supports the essential function of 35 genes, and 26 genes have been targeted with antimalarial drugs. Our analysis identifies 28 essential genes for which no experimental validation was found in the literature. Previous studies have hypothesized the essential function of 15 of the 28 genes. Overall, 13 essential metabolic capabilities had not been previously identified and one of them, which is critical for the production of cardiolipin, is not similar to any human protein (S2 Table). The 28 non-supported predictions require further testing for their validation as potential antimalarial drug targets, as suggested in the iterative cycle of systems biology [11].

The analysis of metabolic tasks identifies the effect of the gene knockouts on the parasite’s growth and suggests that the disruption of the 63 essential genes impair the production of up to 30 biomass precursors (S2 Table). When thermodynamics is taken into account, the number of metabolic tasks impacted by the knockout of one gene increases and it provides additional evidence for the complex interactions between genes, reactions, and metabolites in the metabolic networks. Such is the case of genes required to produce pyrimidines, such as the orotidine 5'-monophosphate decarboxylase, which represents an attractive antimalarial drug target (S2 Table). The knockout of orotidine 5'-monophosphate decarboxylase in iPfa impedes the assembly of pyrimidines, sugar nucleotides, and some DNA nucleotides. This result suggests the thermodynamically dependent function of the metabolic pathways producing phosphorylated nucleotides and sugar nucleotides in *P*. *falciparum*.

We identify eighteen bottleneck metabolites in iPfa, whose intracellular concentrations determine the directionality of a set of reactions and render eight genes essential (Table 2). The bottleneck metabolites are phosphorylated nucleotides, sugar nucleotides, and intermediates in the production of phospholipids. The concentrations of the bottleneck metabolites play a critical role on the directionality of reactions in the cytosol and ER that are mainly involved in the production of phospholipids. Specifically, the genes identified as essential were required to maintain the redox and energy balance in the ER. Additional analysis using the TFA framework helped us quantify the effect of the metabolite concentrations on the reaction directionalities and can guide future metabolomics studies on the malaria parasites (S1 Methods, S1 Dataset). The results of these studies further suggest that there exists in *P*. *falciparum* a thermodynamic dependency between the production of phospholipids, phosphorylated nucleotides, and sugar nucleotides through the concentrations of the bottleneck metabolites. The knowledge of metabolic subsystems that are thermodynamically dependent can be used to design drugs that synergistically target these parts of the metabolism and prevent their metabolic regulation and rise of drug-resistant parasites.

Through the analysis of thermodynamic bottlenecks, we identify two thermodynamically infeasible pathways, i.e. the Kennedy and the pyrimidine biosynthesis pathway, that are functional in *P*. *falciparum* [40]. The presence of substrate channeling between ethanolamine phosphate and PE in the Kennedy pathway and between carbamoyl phosphate and (S)-dihydroorotate in the pyrimidine biosynthetic pathway turns them thermodynamically feasible. Hypotheses on substrate channeling through these pathways have been suggested in other cell types based on the metabolite concentration levels and thermodynamic studies [42, 54, 56]. We demonstrate that network thermodynamics can provide a framework for integrating and testing hypotheses on enzyme mechanisms and their function in metabolic networks [14, 16]. The final validation of substrate channeling in *P*. *falciparum* requires the experimental characterization of the enzymatic mechanisms using techniques such as isotope dilution [18].

The definition and identification of the IMM provide important insight into the nutritional requirements of *P*. *falciparum*. The IMM analysis suggests that *P*. *falciparum* requires at least 23 substrates for growth and 13 of them are indispensable (Table 3). Certain substrates, such as pantothenate [43], hypoxanthine [44] and nicotinate [45], which are provided by default *in vitro*, might be substituted by other substrates to support growth. The ability of substrates to substitute for each other is better understood by considering backbone moieties, which are defined here as molecular substructures shared between substrates that can replace each other to support growth. We find that eight backbone moieties exist in the metabolic network of *P*. *falciparum* and that seven substrates can provide these eight backbone moieties. The analysis of the requirement of the IMM components in a rich medium indicated that *P*. *falciparum* is auxotrophic for the eight backbone moieties (S1 Methods). The malaria parasites might grow on a medium with fewer metabolites that contain the essential backbone moieties. However, not all combinations of substrates support the growth of *P*. *falciparum*. An infeasible combination involves orthophosphate and S-adenosylmethioninamine as the only sources of phosphate and purines. These substrates do not allow the distribution of ATP for the production of phosphorylated nucleotides, sugar nucleotides, sphingomyelin and phospholipids (S1 Methods). The knowledge of nutritional requirements based on molecular substructures will be used to simplify to its maximum extent the medium formulation, design synthetic media [47], and study the uptake mechanism and the metabolic fate of the backbone moieties.

Based on available high-throughput gene knockout data for *P*. *berghei* [57, 58], we expect that most of the genes that TFA predicted to be essential for *P*. *falciparum* development (S2 Table) will also be essential *in vivo*. The study of *P*. *falciparum* metabolism on particular life stages metabolism will allow the identification of additional essential metabolic capabilities and nutritional requirements, such as the need to uptake oleic acid [48]. New methods, such as high-throughput gene knockout [57, 58] and metabolomics techniques, can be applied to *Plasmodium* species and can provide large omics data sets on specific life stages of the malaria infection. iPfa is an ideal scaffold to integrate such context-specific information and to perform integrative studies of *P*. *falciparum* metabolism. A future comparison of *in vivo* knockout high-throughput data and the essentiality analysis on the IMM will advance our understanding of the substrates that are accessible to the malaria parasite in the host. The GEM iPfa should be further developed to keep it up-to-date, and the detailed description of the reconstruction process provided in the S1 Methods serves as a reference for this purpose. Modifications of iPfa will allow the study of context-specific cases, i.e., modeling of the blood stage or liver stage metabolism [59] (S1 Methods).

In conclusion, the results from the TFA of iPfa fill a significant knowledge gap regarding the essential metabolic capabilities and needs of *P*. *falciparum*. The studies on bottleneck metabolites and substrate channeling provide hypotheses for future analysis of the endometabolome and the enzyme mechanisms in malaria parasites. The model, the analysis framework and the results presented here are a valuable resource that can facilitate the ongoing experimental efforts to obtain a better understanding of *P*. *falciparum*’s physiology and identify novel drug targets for antimalarial intervention.

## Materials and methods

### Reconstruction of iPfa

The *P*. *falciparum* 3D7 genome sequence was retrieved from PlasmoDB [60]. The enzymatic functions of the proteins were annotated using the RAVEN Toolbox [24] (S6 Table). A detailed description of the reconstruction process, the software and the data used is provided in the S1 Methods.

### Flux balance analysis (FBA)

FBA is a well-established approach for the computational analysis of large-scale metabolic networks [9, 10]. FBA was performed on iPfa, with the maximization of biomass growth as the objective function.

### Thermodynamics-based flux analysis (TFA)

We integrated the thermodynamic properties of the metabolites and reactions in iPfa in the form of thermodynamic constraints following the systematic approach defined within the framework of Thermodynamics-based Flux Analysis (TFA) [13, 61, 62], which has been also referred to as Thermodynamics-based metabolic Flux Analysis (TMFA) [13] and Thermodynamics-based Flux Balance Analysis (TFBA) [15, 63]. Thermodynamic constraints determine the feasible range of *Δ*_*r*_*G’* and hence reduce the uncertainty in the reaction directionalities and with it the feasible solution space that is characteristic of highly underdetermined problems like the analysis of metabolic networks with Flux Balance Analysis (FBA). These constraints in iPfa accounted for the intracellular conditions, like the pH, the membrane potential and the intracellular concentration ranges of the metabolites (S1 Methods). TFA further allowed the integration of experimentally measured concentration ranges of metabolites [19–22].

### Integration of metabolomics data sets

We created a thermodynamically curated version of iPfa where we allowed the concentration of every intracellular metabolite to vary between 1 μM and 50 mM, which is the physiological range used in similar TFA studies [12, 13]. The concentrations of metabolites outside the parasite cell were allowed to vary between 0.01 μM and 100 mM. We also generated various thermodynamically curated versions of iPfa integrating one-at-a-time each of the ten metabolomics data set considered in this study [19–21]. Nine metabolomics data sets were measured with NMR: eight were obtained from different isolates of *P*. *falciparum* trophozoite-infected red blood cells [20] and one from isolated trophozoites [19]. The remaining data set was measured with LC-MS in isolated trophozoites [21]. The physiological or generic range of concentration (defined above) was considered for a metabolite if no data was available in the metabolomics sets. For the metabolites present in more than one intracellular compartment of iPfa, the same concentration range was defined in all of these compartments. We also generated a combined metabolomics data set [19–22], in which one unique concentration range was calculated for each metabolite appearing in multiple data sets. This unique concentration range comprised all the measured concentration values for that metabolite, i.e. the minimum value and the maximum value measured. Overall, there are absolute concentrations ranges for a total of 61 metabolites [19–22].

We translated the reported values and measurement errors into ranges of concentration expressed in mol/L_cell_. The concentration ranges are integrated within TFA and constrain the allowable Δ_r_G’ range of the reactions in which the metabolites participate and with it the flux ranges of the neighboring reactions. We integrated the metabolomics data sets one-by-one and simultaneously to study the number of bidirectional reactions and the predictions of essential genes. The essential genes varied among data sets, and the data set measured with LC-MS [21] and the combined metabolomics data set allowed the identification of the maximum number of essential genes.

### Gene essentiality per metabolic task

This analysis allowed the identification of the metabolic tasks or biomass building blocks that could not be performed or produced, respectively, upon knockout of the essential genes. The analysis involved two steps: first, the essential genes for biomass growth were identified following standard procedures (S1 Methods). Second, the disruption of each essential gene was applied in iPfa, and the production of each biomass building block was tested. The building blocks that could not be produced individually were identified. An MILP formulation was defined to identify the groups of building blocks that could not be produced at the same time due to stoichiometric requirements. A gene was defined as essential when its knockout led to a specific growth rate slower than 10% of the optimal growth value predicted (S1 Methods). The optimal growth value was the same in all scenarios, i.e. using FBA and TFA. The threshold used does not have any impact on the identification of essential genes and reactions in this study (see Fig SII A and Fig SII B in S1 Methods) since no knockouts led to *in silico* growth reducing phenotypes. No additional filtering was applied to identify essential genes and pairs of genes.

### Comparison of predictions with experimental data

The genes predicted as essential in iPfa with FBA and TFA were compared with experimental information available in the literature. We used primarily experimental data for *P*. *falciparum* (*in vitro*) available in the literature. Information for other *Plasmodium* spp. was also used for comparison. An accuracy score was calculated as (TP+TN)/(TP+TN+FP+FN), where TP (true positive) and TN (true negative) define predictions that correctly simulate growth and non-growth, respectively, based on the available experimental data. While FP (false positive) and FN (false negative) describe predictions that incorrectly simulate growth and non-growth, respectively. The accuracy score served to evaluate the prediction of essential genes. For the predictions with TFA and metabolomics data integrated, an accuracy score of 59% is obtained based on the data available for *P*. *falciparum* in the literature (references reported in S2 Table and previous reviews [64]). Overall in the literature, we found information about the essential function of 71 genes out of the total 325 genes in iPfa. We also evaluated the predictions with the available data from the high-throughput genetic screening of the mouse malaria parasite *P*. *berghei* in the blood stages (PlasmoGEM data) [57, 58]. The accuracy score based on PlasmoGEM data is 51%, and it provides information for 233 orthologous genes out of the total 325 genes in iPfa.

We further tested whether the enzymes predicted as essential in iPfa are similar to any human protein. We define *similarity* between the essential enzymes in iPfa and the human proteome when there exists at least one matching protein in non-redundant human proteome (txid9606) with the standard protein BLAST settings in the bioinformatics software blastp version 2.6.0+ [65]: cutoff value 1E-10, matrix BLOSUM62 [66].

### Sensitivity analysis on the concentration bounds in TFA

We reduced the lower bound ten orders of magnitudes (minimum intracellular concentration of 0.1 fM) and increased the upper bound ten fold (maximum intracellular concentration of 500 mM). We then recalculated the feasible range of *Δ*_*r*_*G’* and tested the gene essentiality of the three genes. These are the three genes that were further identified as essential with TFA using the generic concentration ranges (1 μM—50 mM) with reference to the FBA prediction (S2 Table). The minimum concentration bound required to predict each of the three genes as essential with TFA was identified and reported in Table 2.

### Identification of bottleneck metabolites

This study identified the metabolites responsible for the thermodynamic bottlenecks, which determined the directionality of a set of reactions and allowed the identification of eight genes as essential (Table 2). iPfa was used with generic concentration ranges (1 μM—50 mM) or simultaneous integration of the experimental concentration ranges, and each of the eight genes was knocked out separately (S2 Table). These models were feasible with FBA, but not with TFA. An MILP formulation was defined to search for the minimal number of metabolites whose concentration ranges should be relaxed to make the model feasible in TFA. All the alternative solutions were obtained, and the minimal sets were formed by picking one metabolite from each alternative. The minimal set should involve at least one metabolite from each alternative. The metabolites that were shared among more alternatives appeared in the minimal sets.

### Thermodynamic Variability Analysis (TVA) and inference of reaction directionality

The Gibbs free energy of a reaction (*Δ*_*r*_*G’)* is a measure of its thermodynamic potential and based on the second law of thermodynamics, defines the thermodynamically feasible direction under which the reaction can operate, defined as reaction directionality [14–16]. The *Δ*_*r*_*G’* accounts for the standard Gibbs free energy of a reaction (*Δ*_*r*_*G*^*0*^*’*), which was calculated using the group contribution method (GCM) [61, 62], and for the concentration of the metabolites involved in the reactions. The uncertainty associated with the values of *Δ*_*r*_*G*^*0*^*’* and the metabolite concentrations is expressed within TFA in the form of ranges that ultimately define the feasible range of *Δ*_*r*_*G’* associated with each reaction. The range of *Δ*_*r*_*G’* was known with Thermodynamic Variability Analysis (TVA) [13], which follows the same principles as the Flux Variability Analysis (FVA) [67]. We applied TVA with a requirement of 90% of the optimal growth. When the whole range of *Δ*_*r*_*G’* is negative, then we define the reaction to be unidirectional in the forward direction. When the range of *Δ*_*r*_*G’* is positive and negative, then we define the reaction as bidirectional.

### Substrate channeling integration within TFA

The presence of substrate channeling between two enzymes (E_1_ and E_2_) was simulated within the TFA framework. The reactions R_1_ and R_2_ catalyzed by E_1_ and E_2_ were lumped to define an overall reaction (L = R_1_+R_2_). The overall reaction eliminates the common intermediates and recycled metabolites since they are produced in the first reaction R_1_ and consumed in the second reaction R_2_ and vice versa, respectively. For example, if R_1_ involves the transformation A + B → I + C and R_2_ is defined as I + D → P + B, the final lumped or overall reaction L is defined as A + D → P + C. The overall reaction L does not involve the intermediate metabolite I and the recycled metabolite B. It is important to note that reactions R_1_ and R_2_ should be first defined in the direction that satisfies mass balances and allows flux through the pathway of study. Then the lumped reaction can be formed. For a pathway of study that contains reactions R_1_ and R_2_ the starting and product metabolites are usually A and P, respectively. Then, the reactions R_1_ and R_2_ should be defined as A + B → I + C and I + D → P + B. Other definitions, such as A + B → I + C and P + B → I + D, do not satisfy mass balances. In this study, the reactions of the pyrimidine biosynthesis pathway and the Kennedy pathway were defined to allow production of UMP and PE, respectively. The overall reaction was then allowed to be bidirectional. Thermodynamic properties were calculated for the overall reactions and TFA and Thermodynamic Variability Analysis (TVA) [13] were performed to determine the thermodynamically feasible directionality of the overall reactions. Substrate channeling was suggested when the directionality of the overall reaction allowed flux through the biosynthetic pathway that would be otherwise thermodynamically infeasible. The directionality of the reactions was known from the sign of the range in the *Δ*_*r*_*G’* obtained from TVA.

### Studies on *in silico* minimal medium (IMM)

The IMM analysis was performed following the strategy defined before [3]. Here, TFA was applied with the combined metabolomics data set integrated into iPfa (S1 Methods).

### Identification of groups of substrates that can substitute for each other for growth

This study was performed on the substrates of iPfa to validate their ability to substitute for each other and support growth. Groups of substrates were created based on the IMM analysis. Substrates were grouped if they never appeared in the same IMM and presented a common molecular substructure or backbone moiety. We validated these groups in two steps. First, we tested that iPfa could not grow when all substrates in a group were removed from the *in silico* medium of 52 metabolites identified in the IMM. Second, we tested that under such conditions, the inclusion of each substrate of the group individually allowed simulated growth in iPfa. This analysis was also performed in the rich medium of 236 substrates to validate the groups of substrates that contained the three backbone moieties (the sources of carbon, phosphate, and purine).

The tools and methods used are described in more detail in S1 Methods.

## Supporting information

S1 Methods Describes in more detail the tools and methods used in this study. (PDF)

S1 Table Gap-filling reactions and alternatives that determine the thermodynamic feasibility of the metabolic tasks in iPfa and the *in silico* rich medium. ^1^Gap-filling reactions are defined with their R-5 digit identifier as obtained from the KEGG database. Transport reactions are marked with T_. Cellular compartments are defined with the reaction name: _r, endoplasmic reticulum; _c, cytosol; _m, mitochondrion; _a, apicoplast. Note that the same gap-filling reaction can be suggested for more than one metabolic task.
(XLSX)

S2 Table Single essentiality predictions in iPfa. ^1^Genes predicted as essential for iPfa growth in the *in silico* rich medium (composed of 236 substrates, S1 Methods). ^2^Life stage of the malaria infection at which the experiment reported in the literature was performed: B, blood stages; L, liver stage; M, mosquito stage. The experimental description of the gene is defined in parenthesis: (e) essential; (gr) growth reducing; (ne) non-essential. ^3^Impact of the gene knockout on the metabolic tasks of iPfa is explainable based on the annotated function of the gene. ^4^This gene is involved in an essential pathway and is thus presumed to be essential. ^5^None of the reactions associated with this gene is single essential; the gene is essential when two or more associated reactions are knocked out. ^6^We define *similarity* between the essential enzymes in iPfa and the human proteome when there exists at least one matching protein in non-redundant human proteome (txid9606) with the standard protein BLAST settings in the bioinformatics software blastp version 2.6.0+ [65]: cutoff value 1E-10, matrix BLOSUM62 [66].
(XLSX)

S3 Table Synthetic lethal pairs in iPfa. ^1^Pairs of genes predicted as essential for iPfa growth with TFA and the MS data (2012, Vo Duy *et al*.) in the *in silico* rich medium (composed of 236 substrates, S1 Methods).
(XLSX)

S4 Table List of reactions that become unidirectional with TFA. ^1^Reaction I.D. in iPfa. Cellular compartments are defined after “_” in the reaction I.D.: (_c) cytosol, (_m) mitochondrion, (_r) endoplasmic reticulum, (_e) extracellular. Transport reactions are marked with T_. ^2^Values of the standard Gibbs free energy of reaction (*ΔG*_*0*_*'*) calculated with the group contribution method (GCM), as explained in the S1 Methods. Note that *ΔG*_*0*_*'* does not determine the reaction directionality. ^3^Reaction directionalities determined with Thermodynamic Variability Analysis (TVA). ^4^The reactions R02057_r, R01468_r, R02038_r, and T_c_to_r_C00189 are unidirectional (forwards) using TVA and generic concentration ranges, or blocked using TVA and metabolomics data (see Table 2).
(XLSX)

S5 Table Single essentiality predictions in iPfa among the alternative *in silico* minimal media (IMMs). ^1^Genes predicted as essential for iPfa growth with TFA and the MS data (2012, Vo Duy *et al*. [21]) in the alternative IMMs (10032 alternative IMMs, each composed of 23 substrates, see Table 2). ^2^Genes commonly essential in all alternative IMMs. ^3^If more than one reaction is associated with the gene, one enzyme name was chosen.
(XLSX)

S6 Table Genes of *P*. *falciparum* and associations with K-Orthology groups obtained with the RAVEN Toolbox. ^1^Gene IDs were obtained from PlasmoDB. ^2^K-ID or Orthology groups were obtained from the KEGG database. ^3^The E-value denotes the goodness of the annotation to the assigned metabolic function. The annotation process is described in detail in S1 Methods.
(XLSX)

S7 Table Localization evidence and scores for the genes in the draft metabolic network of iPfa. ^1^Localizations from the ApiLoc database. Intracellular compartments are defined by letters, such as c, cytosol; m, mitochondrion; a, apicoplast; er, endoplasmic reticulum; fv, food vacuole; e, extracellular space/parasitophorous vacuole; n, nucleus; ag, Golgi apparatus.
(XLSX)

S1 Dataset Ranking of metabolites based on the *Reduction of Uncertainty* (*RoU*) in the Gibbs free energy of the reactions (*Δ*_*r*_*G’*). The concentration range of each metabolite was integrated separately in iPfa following two approaches. In the first approach, the concentration of the metabolite was integrated at-a-time in all compartments of iPfa where the metabolite appears (sheet S1 A and sheet S1 B in S1 Dataset). In the second approach, the concentration of the metabolite was integrated separately in each compartment of iPfa where the metabolite appears (sheet S1 C and sheet S1 D in S1 Dataset). The RoU in the *Δ*_*r*_*G’* was calculated in both approaches. The reactions in iPfa were ranked based on two ranking criteria: the number of reactions impacted by each metabolite (sheet S1 A and sheet S1 C in S1 Dataset), and the global RoU (sheet S1 B and sheet S1 D in S1 Dataset) as defined in the S1 Methods. Note: we consider that a metabolite impacts a reaction if the RoU in *Δ*_*r*_*G’* is higher than 0.1%.
(XLSX)
