# Supplementary material for: Bioenergetics-based modeling of Plasmodium falciparum metabolism reveals its essential genes, nutritional requirements, and thermodynamic bottlenecks
Source: PLoS Comput Biol. 2017 Mar 23;13(3):e1005397. doi: 10.1371/journal.pcbi.1005397 (PMC5363809; doi:10.1371/journal.pcbi.1005397)
Supplement: S1 Methods — (PDF) [file pcbi.1005397.s001.pdf]

# Supporting Information

## Chiappino-Pepe *et al.*

**S1 Methods**

**Part 1. Reconstruction process of iPfa**

**Steps in the reconstruction process of iPfa.** The reconstruction process of iPfa involved six main steps: (i) generation of a draft metabolic network with the RAVEN Toolbox [1] (S6 Table) and its curation; (ii) compartmentalization of the metabolic network, which involved the assignment of enzymes to intracellular compartments and the definition of transport reactions between compartments (S7 Table); (iii) definition of biomass composition in the form of a biomass reaction; (iv) definition of uptakes and secretions of the parasite cell, which involved the definition of a broad range of potential substrates that are likely to be accessible to the parasites from the host cells and the blood serum [2, 3]; (v) thermodynamic curation of the model, as performed previously [4-6]; and (vi) addition of missing metabolic capabilities, so called gap-filling reactions, that are necessary to simulate growth (S1 Table). We also integrated experimentally measured uptake and secretion rates, i.e., for glucose [7], L-isoleucine [8] and lactate [7].

**Generation of a draft metabolic network.** We retrieved the protein sequences (FASTA files) of *P. falciparum* 3D7 from PlasmoDB [9], which is based on the genome sequencing performed in 2002 [10], and annotated the enzymatic functions to the proteins with the RAVEN Toolbox [1]. The generation of the draft metabolic network was determined by two factors: the parameters specified in the annotation process (i.e. cut-off value and minimal score ratios, as defined below); and the associations between orthologous groups (5 digit K-ID), proteins and reactions that were obtained from the KEGG database [11].
The annotation process, i.e., matching between the protein sequences and the orthologous groups, was performed with the function getKEGGModelForOrganism in the RAVEN Toolbox [1]. Three main input parameters were specified to define the goodness of the annotations: the cut-off value of $10^{-15}$, the minimal score ratio of 0.8 and the minimal score ratio for a knocked out gene of 0.3. The output of this annotation process is summarized in S6 Table.
The versions of the software and the data used in the reconstruction process were: version 1.07 of the RAVEN Toolbox, version 11.1 of the protein FASTA files/protein sequences for *P. falciparum* 3D7 from PlasmoDB, and the version of KEGG as of July 2014.

We evaluated and curated the gene annotations obtained from RAVEN with the experimental evidence on gene and/or enzyme characterization in *P. falciparum* available in the literature. In this curation process, we also assigned unique metabolite identifiers to some molecules, such as the alpha and beta isoforms of the monosaccharide D-glucose and D-fructose, and the diacylglycerol with 1,2-diacyl-sn-glycerol. In addition, we corrected the reactions for cofactor specificity when information was available. For instance, the quinone participating in the electron transport chain in the mitochondrion was assigned to be ubiquinone-8 [12].

For instance, based on recent findings [13], we incorporated in iPfa the pyruvate dehydrogenase activity of the mitochondrial BCKDH complex. The BCKDH-PDH function has been experimentally characterized in the rodent malaria parasite *P. berghei* [13], and has been annotated to the genes defined in Table SI.

We inferred the function of the mitochondrial BCKDH-PDH in *P. falciparum* using as reference the experimental characterization of this complex in *P. berghei.*

We performed a bidirectional homology measurement of the protein sequences in *P. falciparum* and *P. berghei* with the RAVEN Toolbox [1] and we have inferred the orthologous genes in *P. falciparum* with the enzymatic function of BCKDH-PDH complex (Table SII). Based on of the BLASTp, the sequence similarity between the orthologous sequences is lower than 1E-172 for the four genes involved in the BCKDH-PDH complex, which suggests this function is also present in *P. falciparum*.

**Table SI.** Experimental annotation of mitochondrial BCKDH-PDH in *P. berghei* as defined by Oppenheim *et al.* [13].

| **E.C. number** | **Experimental annotation** | **Gene *P. berghei*** (updated ID) | **Orthologous *P. falciparum* gene** (suggested in [13]) |
|---|---|---|---|
| 1.2.4.4 | Branched-chain alpha-ketoacid dehydrogenase E1 alpha (BCKDHNE1a) | PBANKA_1411100 | PF3D7_1312600 |
| 1.2.4.4 | Branched-chain alpha-keto acid dehydrogenase E1 beta (BCKDHNE1b) | PBANKA_1104200 | PF3D7_0504600 |
| 2.3.1.168 | Branched-chain alpha-keto acid dehydrogenase E2 (BCKDHN E2) | PBANKA_0402300 | PF3D7_0303700 |
| 1.8.1.4 | Lipoamide dehydrogenase E3 (BCKDHNE3) | PBANKA_1446900 | PF3D7_1232200 |

**Table SII.** Result from homology measurement between protein sequences in *P. falciparum* and *P. berghei* performed with the RAVEN Toolbox [1] in this study.

| **Gene *P. berghei*** | **Inferred orthologous *P. falciparum* gene** | **E-value** | **Identity** | **Alignment Length** |
|---|---|---|---|---|
| **PBANKA_1411100** | PF3D7_1312600 | 0 | 83.95 | 430 |
| **PBANKA_1104200** | PF3D7_0504600 | 0 | 86.09 | 338 |
| **PBANKA_0402300** | PF3D7_0303700 | 1.00E-172 | 57.94 | 447 |
| **PBANKA_1446900** | PF3D7_1232200 | 0 | 79.8 | 505 |

**Compartmentalization.** The compartmentalization process of iPfa involved the localization of enzymes in five intracellular compartments (i.e. the cytosol, the apicoplast, the mitochondrion, the endoplasmic reticulum and the nucleus) and the definition of transport reactions between these compartments.

Nearly 25% of the enzymes in iPfa were localized based on experimental evidence. The information on experimental localization of enzymes in *P. falciparum* was obtained from the online databases ApiLoc [14] (last time updated in 2011) and from MPMP [15] (last time accessed in March 2015). The remaining 75% of the enzymes were assigned to the intracellular compartments based on localization scores. These scores compare the protein sequences to be localized with the amino acid sequences that target the proteins to the specific intracellular compartments, such as the bipartite targeting sequence for the apicoplast in *P. falciparum* [16]. The software used to generate the localization scores were TargetP [17] (version 1.1), MitoProt II [18] (version 1.101), and ApicoAP [19] (version 2). The input to these software was the FASTA file obtained from PlasmoDB (version 11.1, strain 3D7) containing the information on the protein sequences of *P. falciparum,* as

described in the generation of a draft metabolic network. The experimental localization and localization scores obtained for the enzymes and the associated genes in iPfa are summarized in S7 Table.

*P. falciparum* has other intracellular compartments (e.g. digestive vacuole, Golgi apparatus) that were not explicitly included in iPfa. These compartments were *a priori* considered in the reconstruction of iPfa. However, very few enzymes in iPfa (i.e., four enzymes in the digestive vacuole and one enzyme in the Golgi apparatus) were experimentally localized in these compartments (S7 Table). We then decided to include the corresponding reactions in the cytosol.
For example, some of the reactions that occur in the digestive vacuole, such as the hemoglobin digestion/degradation, are included in iPfa and located in the cytosol. Other processes in this organelle, such as the accumulation of hemozoin and $Ca^{2+}$, are not considered in this study since a (quasi)-steady state condition for the metabolites should be satisfied within flux balance and thermodynamics flux analyses.

Intracellular transport reactions allow the exchange of metabolites between intracellular compartments. Under the absence of information or experimental evidence about the transport reactions in *P. falciparum* [20], we defined transporters for all cytosolic metabolites that were also present in other intracellular compartments, only if these metabolites fulfilled three conditions: they were not phosphorylated, they did not present a Coenzyme A (CoA) and neither a [acyl-carrier protein] (ACP) moiety attached (following the same considerations that had been applied in previous studies, such as ToxoNet1 [21]). In these cases, the transport mechanism considered was simple diffusion. We allowed the transport of phosphorylated molecules that has been previously characterized in *P. falciparum*, such as the transport of phosphoenolpyruvate and glycerone phosphate into the apicoplast [22].

**Biomass definition.** The biomass reaction in a genome-scale metabolic model (GEM) comprises all major biomass components of the cell. We defined in the biomass reaction of iPfa five major biomass macromolecule groups, i.e., *DNA*, *RNA*, *Protein*, *Lipids* and *Carbohydrates and others*. We defined the metabolic precursors or biomass building blocks and their quantity in each macromolecule group based on available experimental data in the literature.
The frequency of DNA and RNA nucleotides and amino acids in *P. falciparum* has been experimentally characterized and reported [23]. In the same way, we obtained the frequency of the lipid components in the parasite, Table 1 in [24] and Table 2 in [25]. The distribution of free fatty acids was estimated from available experimental results [25, 26]. The biomass precursors for which no experimental quantification in *P. falciparum* was found were grouped in *Carbohydrates and others*. They represent nucleotide sugars, polyamines, prosthetic groups, coenzymes and ions, which are commonly defined as essential biomass components [27]. In this case, we calculated their frequency assuming a distribution proportional to the number of carbons present in their molecular composition. We did not define other putative biomass precursors like glycoproteins, which are part of the GPI-anchors, because detailed knowledge about their production from the sugars nucleotides was not found for *P. falciparum*. Instead their precursors, i.e. the nucleotide sugars, where defined as biomass components.

When it was not specified, the frequencies obtained from the different sources were assumed to be in %-mol/mol. All frequencies were normalized to calculate the final

stoichiometric coefficients, as defined in standard reconstruction protocols [28]. Growth associated maintenance (GAM), which accounts for non-metabolic processes, was calculated based on the biomass composition, as commonly performed [28].

**Definition of the available substrates in iPfa: uptakes and secretions.** iPfa has an *in silico* rich medium composed of 236 potential substrates. The medium in iPfa was primarily compiled based on the general assumption about the transportability of the metabolites in the cytosol: unless evidence was found to define a specific transport mechanism, we allowed the transport of all molecules that do not incorporate phosphate, coenzyme A (CoA) and acyl-carrier proteins (ACP) in their molecular structures, and we assumed that transports occur through passive diffusion. We further included in the medium metabolites that were not present in the cytosol of the draft metabolic network, and that are part of the the RPMI 1640 media formulation [2] and the serum composition [3]. These metabolites were accounted in our gap-filling approach to identify precursors of the biomass building blocks. Such an approach allowed the definition of precursors like pantothenate, thymidine and succinate in the *in silico* rich medium of iPfa.

In addition, iPfa integrates available experimental data on uptake and secretion rates in *P. falciparum* trophozoites. The efflux values integrated included the maximum uptake rate of L-isoleucine (set to 0.053 mmol/h-gDW cell, as calculated from the value 553 ± 27 μmol/($10^{12}$ cells per hour) [8]) and of D-glucose (set to 0.62 mmol/h-gDW cell, as calculated from the value 120 ± 34 μM glucose/($10^9$ parasitized red blood cells per 24 hour) [7]), and the maximum secretion rate of lactate (set to 0.76 mmol/h-gDW cell, based on the value 143 ± 47 μM lactate/($10^9$ parasitized red blood cells per 24 hour) [7] and consistent with previous data [29, 30]). To calculate these values we assumed a cell dry-weight for *P. falciparum* of $1.05 \bullet 10^{-11}$ gDW/cell [23], which represents around 30% of the erythrocyte dry-weight [31].

The predicted growth rate in iPfa is 0.16 $h^{-1}$ after integration of the L-isoleucine uptake rate. This value is comparable with the growth rates derived from the doubling time in *P. falciparum* trophozoites [32]. The number of malaria parasites in the blood stages increases exponentially [33], hence Eq 1 can be applied to estimate the growth rate of *P. falciparum* in the blood stages.

$$gr = \frac{\ln\left( N(t) / N(0) \right)}{t} \qquad (1)$$

where N(t) and N(0) is the number of cells at time t and time 0 and gr is the growth rate. The coefficient of N(t) and N(0) is two in the calculation of the doubling time.

For a doubling time of 12.3 h [32], the specific growth rate of *P. falciparum* in the blood stages is around 0.06 $h^{-1}$. In this study, we assumed a broader physiological range of growth rate, i.e. between 0.05 and 0.14 $h^{-1}$. This range accounts for the development of 14 and 32 merozoites within 24 or 48 hours as observed in the *in vitro* cultivation of *P. falciparum* during one cycle of erythrocyte infection.

**Thermodynamic curation of iPfa. Data integrated: pH, membrane potential and concentration data. Statistics.** The Gibbs free energy of a reaction ($\Delta_r G'$) is a measure of

its driving force and determines thermodynamically feasible direction under which the reaction can operate, defined as reaction directionality [34-36]. We integrated the thermodynamic properties of the metabolites and reactions in iPfa in the form of thermodynamic constraints following the systematic approach defined within the framework of Thermodynamics-based Flux Analysis (TFA) [4-6]. Thermodynamic constraints determine the feasible range of $\Delta_r G'$ and hence reduce the uncertainty in the reaction directionalities and with it the feasible solution space that is characteristic of highly underdetermined problems like the analysis of metabolic networks with Flux Balance Analysis (FBA). These constraints in iPfa accounted for the intracellular conditions, like the pH, the membrane potential and the intracellular concentration ranges of the metabolites. TFA further allowed the integration of experimentally measured concentration ranges of metabolites [37-40].

We defined a pH of 7.3 in the cytosol, based on the pH value and the membrane potential reported in [41, 42]. Under the absence of information, we assumed the same pH value in the endoplasmic reticulum and the nucleus. We estimated a pH of 7.5 in the mitochondrion of *P. falciparum*, based on measurements in *Saccharomyces cerevisiae* [43] and the similar mitochondrial membrane potential that both organisms present, as argued below and as it has been hypothesized before for similar pathogens like *Trypanosoma cruzi*: "it is able to build up and retain a membrane potential of a value comparable with that of mammalian mitochondrion" [44]. We defined a pH value of 6.8 in the extracellular medium, i.e., the parasitophorous vacuole or *in silico* rich medium in iPfa, which was obtained from interpolation of Fig 9 in [42] for a ±95 mV membrane potential, and it is consistent with the statement in [45] that the extracellular pH should be higher than 6.5. We defined a membrane potential of ±95 mV for the extracellular membrane of the parasite, as measured in [42]. Based on the experimental studies in *P. berghei* and *P. yoelii* trophozoites [46, 47] and *Toxoplasma gondii* tachyzoites [48], the membrane potential in the mitochondrion of *Plasmodium* parasites should be higher than ±150 mV. We assumed a membrane potential of ±180 mV for the mitochondrial membrane, which is the value in *S. cerevisiae* [49].

We created a thermodynamically curated version of iPfa where we allowed the concentration of every metabolite to vary between 1 μM and 50 mM, which is the physiological range used in similar TFA studies [4, 50]. The extracellular metabolite concentrations were allowed to vary between 0.01 μM and 100 mM. We also generated various thermodynamically curated versions of iPfa integrating one-at-a-time each of the ten metabolomics data set considered in this study [37-39]. Nine metabolomics data sets were measured with NMR: eight were obtained from different isolates of *P. falciparum* trophozoite-infected red blood cells [38] and one from isolated trophozoites [37]. The remaining data set was measured with LC-MS in isolated trophozoites [39]. The physiological range of concentration (defined above) was considered for a metabolite, if no data was available in the metabolomics sets. For the metabolites present in more than one intracellular compartment of iPfa, the same concentration range was defined in all of these compartments. We also generated a combined metabolomics data set [37-40], where one unique concentration range was calculated for each metabolite appearing in multiple data sets. This unique concentration range comprised all the measured concentration values (Fig SI A and B). The absolute concentration values and the concentration ratios of the metabolites determine the final $\Delta_r G'$. For example, the range of the ratio NADP/NADPH in the combined metabolomics data set varies between 1.6 and 6.0 mol/$L_{cell}$ / mol/$L_{cell}$.

A

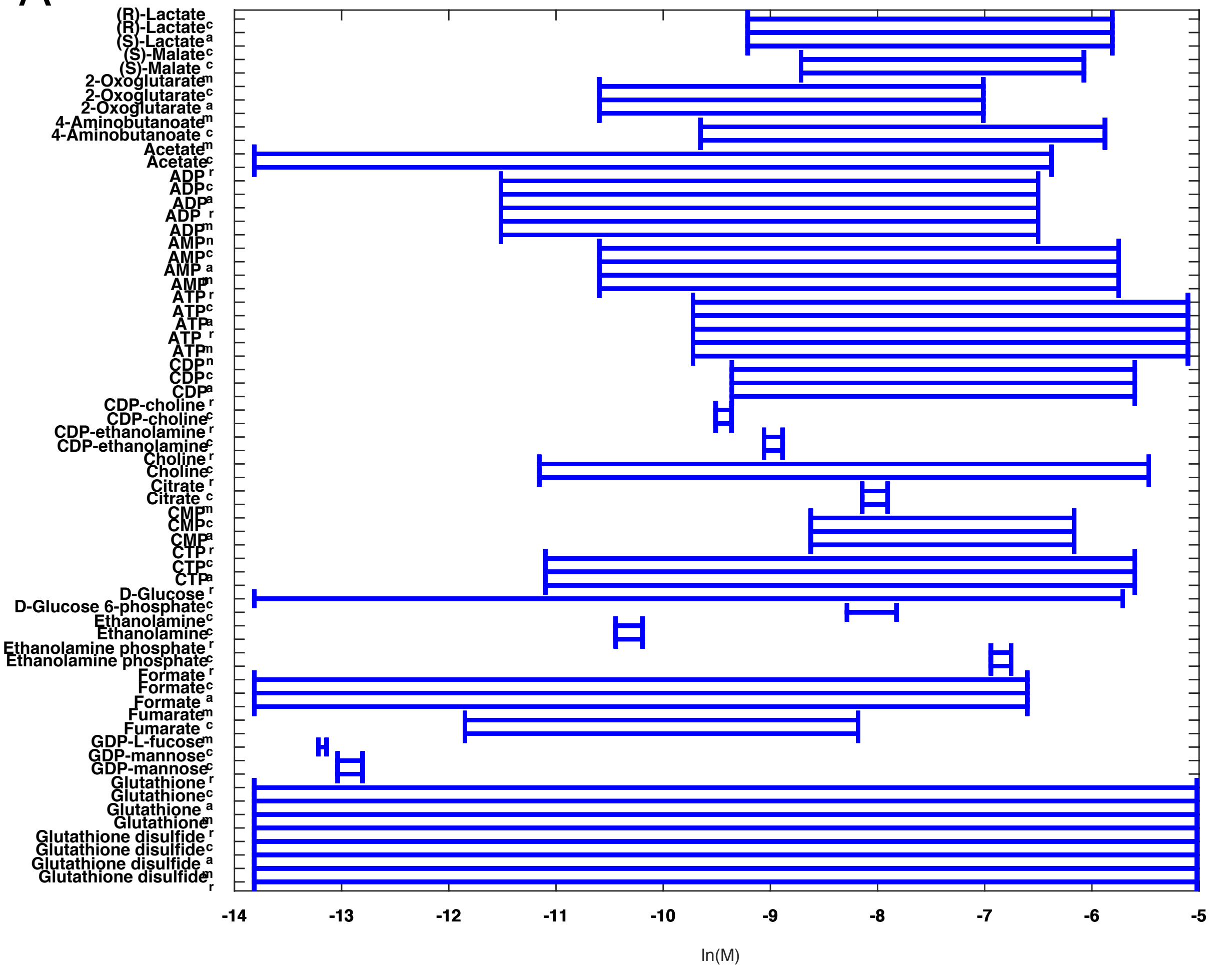
(R)-Lactate
(R)-Lactate c
(S)-Lactate a
(S)-Malate c
(S)-Malate c
2-Oxoglutarate m
2-Oxoglutarate c
2-Oxoglutarate a
4-Aminobutanoate m
4-Aminobutanoate c
Acetate m
Acetate c
ADP r
ADP c
ADP a
ADP r
ADP m
AMP n
AMP c
AMP a
AMP m
ATP r
ATP c
ATP a
ATP r
ATP m
CDP n
CDP c
CDP a
CDP-choline r
CDP-choline c
CDP-ethanolamine r
CDP-ethanolamine c
Choline r
Choline c
Citrate r
Citrate c
CMP m
CMP c
CMP a
CTP r
CTP c
CTP a
D-Glucose r
D-Glucose 6-phosphate c
Ethanolamine c
Ethanolamine c
Ethanolamine phosphate r
Ethanolamine phosphate c
Formate r
Formate c
Formate a
Fumarate m
Fumarate c
GDP-L-fucose m
GDP-mannose c
GDP-mannose c
Glutathione r
Glutathione c
Glutathione a
Glutathione m
Glutathione disulfide r
Glutathione disulfide c
Glutathione disulfide a
Glutathione disulfide m
r
-14
-13
-12
-11
-10
-9
-8
-7
-6
-5
ln(M)

B

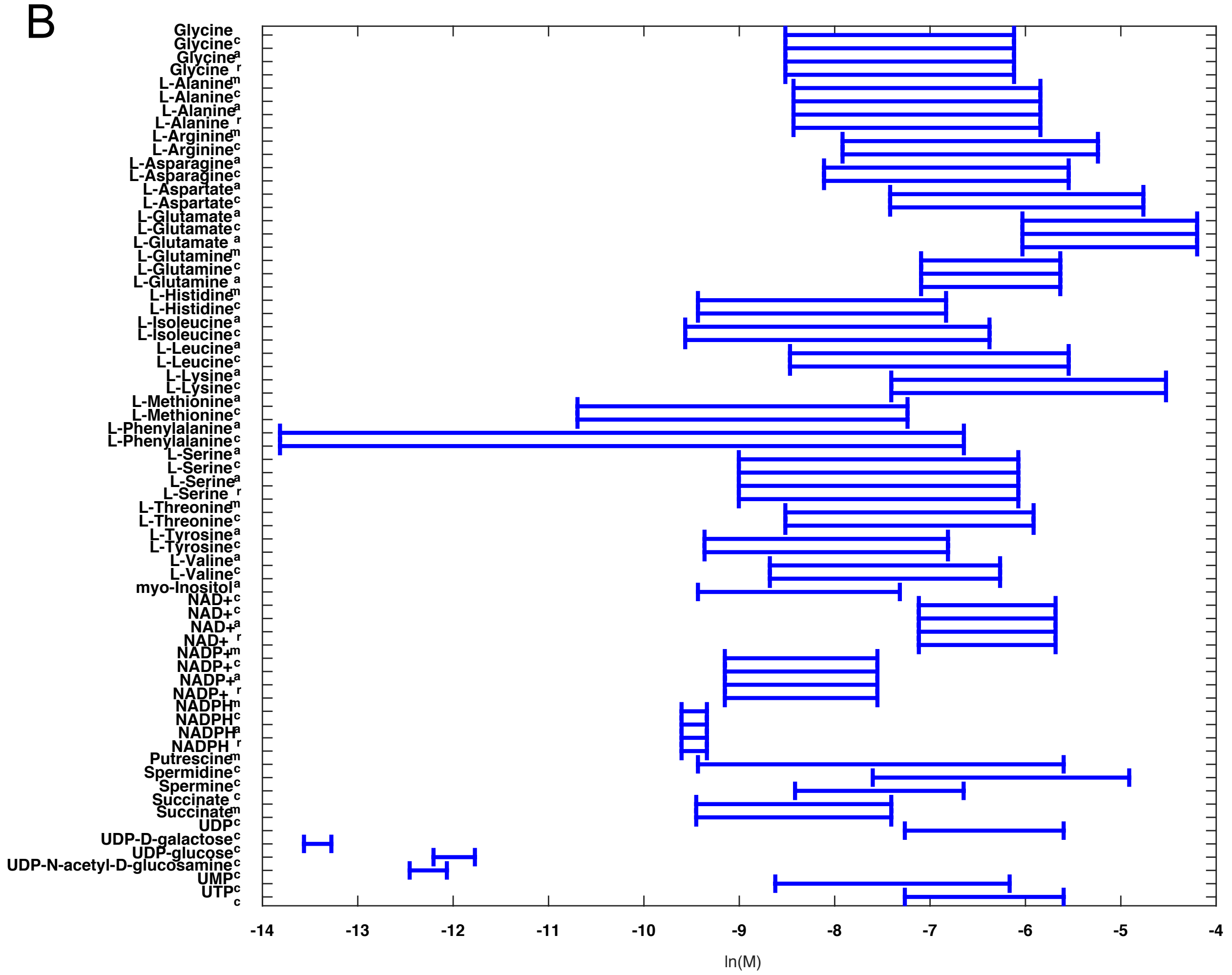


**Fig SI A and B. Absolute concentration ranges in logarithmic scale (LN of mol/L of cell) of the metabolites in the combined metabolomics data set.**

We calculated the thermodynamic properties of the compounds in iPfa using the group contribution method (GCM) [5, 6]. In this study, the R groups of large fatty acids and phospholipids were not specified and hence their thermodynamic properties were not considered.

In addition, we checked all reactions for correct atom balancing at the pH considered in the corresponding intracellular compartments. Overall in iPfa, thermodynamic properties were calculated for 70% of the total number of enzymatic and transport reactions and nearly 84% of them did not present pre-assigned directionality. The pre-assigned directionalities in iPfa are based on the directionalities defined in the KEGG database, which were reviewed using the enzyme database BRENDA [51]. We additionally prevented the hydrolysis reactions (EC 3.-.-.-) for which no thermodynamic properties could be calculated to occur in the reverse direction. Note that thermodynamic constraints affect also the reactions for which no thermodynamic properties are calculated. For instance, the directionality of the reactions with thermodynamic information can determine the directionality of the neighboring reactions. This is an effect of the propagation of mass and

thermodynamic constraints due to the connectivity of the metabolic network and the growth requirement, as discussed in previous studies on network thermodynamics [34].

**Metabolic tasks and gap-filling.** Metabolic tasks are defined in this study as the production of biomass building blocks [1, 21]. The draft metabolic network of iPfa did not contain all the necessary reactions that allowed simulation of growth. This is a common situation in the reconstruction process of GEMs [28] and requires the inclusion of reactions without gene association in a process called gap-filling [52]. We evaluated each metabolic task in iPfa and performed gap-filling when a metabolic task was not feasible, i.e. when a biomass building block could not be produced in iPfa. We developed a gap-filling approach based on a mixed-integer linear programming (MILP) formulation to look for alternative groups of minimal number of reactions (borrowed from another metabolic network) that determined the feasibility of all metabolic tasks. This formulation allows the integration of thermodynamic constraints.
In the draft metabolic network of iPfa 24 of the 73 total metabolic tasks were not feasible. We used ToxoNet1 [21] to gap-fill 15 of these metabolic tasks. The remaining 9 metabolic tasks were gap-filled with reactions from the KEGG-based metabolic network as of 2015, which comprises all to date known enzymatic reactions in any organism [11]. These gap-filling reactions and alternatives are reported in S1 Table.

**Part 2. Analyses of iPfa**
**Studies on essential genes and reactions.** Following standard procedures, single reaction and gene knockout simulations were performed in iPfa; the reactions and genes were deleted one-by-one and growth was simulated. In a similar fashion, double gene knockouts implied the pairwise deletion of non-single essential genes. Reactions or genes were defined as essential when simulation of their knockout resulted in a growth rate smaller than 10% of the optimal predicted value, i.e., 0.16 $h^{-1}$. This value corresponds to a 90% reduction of the maximum specific growth rate predicted with iPfa when experimental efflux values are integrated (see the step on the definition of uptakes and secretions in the reconstruction process of iPfa to learn about the specific growth rate of *P. falciparum*). The results of gene and reaction essentiality in iPfa reported in S2 Table and S3 Table were obtained using the *in silico* rich medium composed of 236 metabolites.

The optimal predicted growth value was the same in all scenarios, i.e. using FBA and TFA. The threshold of 10% used to identify essential genes and pairs of genes does not have any impact on the identification of essential genes and reactions in this study (see Fig SII A and B), since no knockouts led to *in silico* growth reducing phenotypes. No additional filtering was applied to identify essential genes and pairs of genes.

The comparison of single gene and reaction essentiality allowed to understand what enzymes of iPfa rendered the genes essential (S2 Table). Two classes of genes were identified based on the essentiality of its associated reactions. The first class involves genes that are essential because one (or more) of its encoded enzymes is (are) alone indispensable for growth. In this case, the gene is essential *in silico* and one (or more) of its associated reactions is (are) also essential *in silico*. The second class involves genes that are essential because a group of its encoded enzymes is indispensable (and not any of its encoded enzymes alone). In this case, the gene is essential *in silico* and none of its associated reactions appears as single essential, which happens when the gene-to-reaction associations is not one-to-one. In S2 Table the reactions and enzymes that are responsible for the essentiality of the *in silico* essential genes are presented. This

information is relevant because genes are normally classified as essential or non-essential based on the outcome from experimental gene knockouts, but medical therapies are normally designed to target essential enzymes in the pathogens. Thus, it is of interest to know, which of the enzymes encoded by the essential gene performs the essential function in the metabolic network of the pathogen.

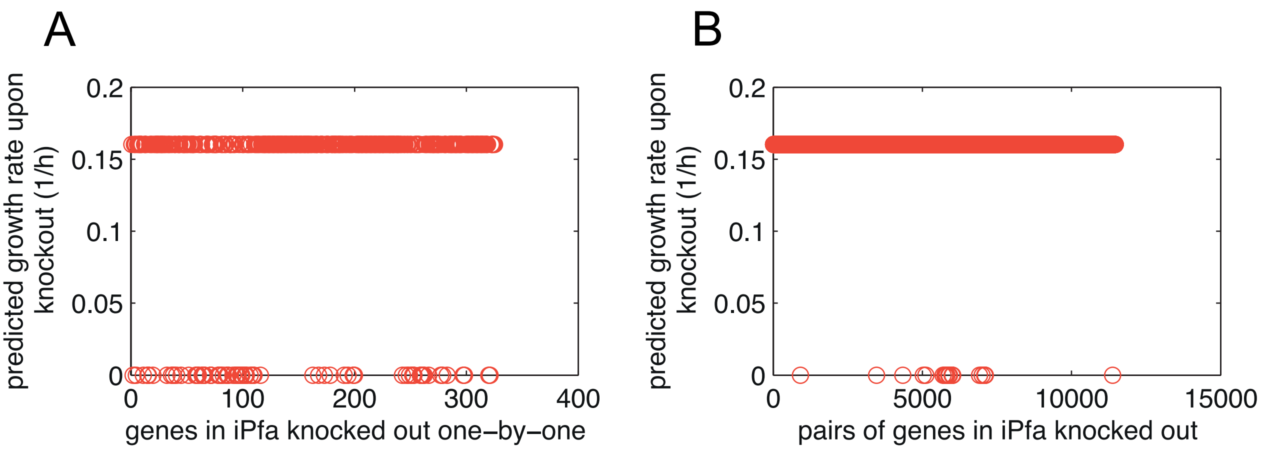


**Fig SII A and B.** Predicted phenotype upon single (A) and double (B) gene knockout in iPfa. Predictions made with TFA with the combined metabolomics data set integrated.

**Gene essentiality per metabolic task.** This analysis allowed the identification of the metabolic tasks or biomass building blocks that could not be performed or produced, respectively, upon knockout of the essential genes. In addition to the analysis described in *Methods*, a MILP formulation was defined to identify the groups of building blocks that could not be produced at the same time due to stoichiometric requirements. The analysis was performed with FBA and TFA.

The study of the metabolic tasks using TFA provides additional understanding of the effect of the knockouts. TFA constrains further the metabolic network and thus there are less alternative pathways to fulfill a metabolic task. In turn, when thermodynamics is taken into account, the number of metabolic tasks impacted by the knockout of one gene might increase. This analysis also suggests a thermodynamic dependency between the metabolic subsystems.

**Ranking of metabolites based on the *Reduction of Uncertainty* (*RoU*) in the Gibbs free energy of the reactions ($\Delta_r G'$).** FBA defines mass balance constraints for each metabolite in the GEM and solves a linear problem to determine the intracellular fluxes. Thus, the FBA of a genome-scale model with a higher number of reactions than metabolites is underdetermined and has an inherent uncertainty in its solution, referred to as feasible solution space [53]. TFA, with the integration of thermodynamic constraints reduces this space and allows only the thermodynamically feasible solutions. However, there is still an uncertainty or feasible range in the fluxes and other thermodynamic properties like the $\Delta_r G'$. The range in the $\Delta_r G'$ is directly related with the concentration ranges of the metabolites participating in the reaction.

In iPfa a reduction of uncertainty in the concentration ranges of the metabolites and hence in the $\Delta_r G'$ of the reactions was achieved by integrating metabolomics data (see the

description of the thermodynamic curation of iPfa to know the concentrations used). Here, studies were performed on iPfa to identify the metabolites from the metabolomics data sets used that determined the highest reduction of uncertainty in the $\Delta_r G'$, referred to as Reduction of Uncertainty (RoU) and defined in Eq 2. The RoU identifies which reactions are impacted individually by each metabolite concentration, to which extent are these reactions impacted and hence which are the most impacting metabolites (S1 Dataset). This analysis is independent of the studies on bottleneck metabolites (Table 2, Materials and Methods), and allows a ranking of the metabolites based on a different metric. Consistent with experimental observations [54], the results of both studies indicate that the phosphorylated nucleotides appear among the most impacting metabolites.

The RoU in $\Delta_r G'$ was calculated for a reaction i by comparing its range of $\Delta_r G'$ when physiological ranges were assumed for all metabolites (defined as $\Delta\Delta_r G'_{ref,i}$ in Eq 1) with its range of $\Delta_r G'$ when the experimental concentration range of one metabolite j was integrated ($\Delta\Delta_r G'_{[met\ j],\ i}$). A global RoU was then calculated by adding the $RoU_i$ of all the reactions in iPfa to define the overall impact of each metabolite (S1 Dataset).

$$RoU_i = \frac{\Delta\Delta G'_{ref,i} - \Delta\Delta G'_{[met,j],i}}{\Delta\Delta G'_{ref,i}} \qquad (2)$$

The metabolite concentration ranges of the combined metabolomics data sets were used for this study (see the description of the thermodynamic curation of iPfa to know the experimental data that comprised this set). The range of $\Delta_r G'$ was obtained with Thermodynamic Variability Analysis (TVA) [4], which follows the same principles as the Flux Variability Analysis (FVA) [55]. In TVA a growth rate of iPfa between the 80% of its optimal value and its optimal value, i.e., between 0.14 and 0.16 $h^{-1}$, was required (see the step on the definition of uptakes and secretions in the reconstruction process of iPfa to understand the source of these values).

This study falls within the scope of a well developed framework called Thermodynamics-based Metabolite Sensitivity Analysis (TMSA) that has been developed by A. Kiparissides & V. Hatzimanikatis [56] for the ranking of metabolites that reduce the uncertainty of different parameters in the metabolic networks and allow a better definition of the internal states of the cells.

**Studies of bottleneck metabolites and reactions impacted.** This study identified the metabolites responsible for the thermodynamic bottlenecks, which determined the directionality of a set of reactions and allowed the identification of eight genes as essential (Table 2). iPfa was used with generic concentration ranges (1 μM - 50 mM) or simultaneous integration of the experimental concentration ranges, and each of the eight genes was knocked out separately (S2 Table). These models were feasible with FBA, but not with TFA. A MILP formulation was defined to search for the minimal number of metabolites whose concentration ranges should be relaxed to make the model feasible in TFA. All the alternative solutions were obtained, and the minimal sets were formed by picking one metabolite from each alternative. The minimal set should involve at least one metabolite from each alternative. The metabolites that were shared among more alternatives appeared in the minimal sets.

**Table SIII A. Unique set of reactions impacted after integration of the bottleneck metabolite concentration ranges associated to the gene PF3D7_1415700.**

| Reaction ID[1] (Reaction directionalities[2]) |
|---|
| R01280_c (B) |
| R00371_c (B) |
| R01281_c (B) |
| R00371_r (B) |
| R01281_r (B) |
| T_c_to_r_C00037 (B) |
| T_c_to_r_C03508 (B) |
| T_c_to_e_C03508 (B) |
| R02055_r (F) |
| T_c_to_r_C00065 (F) |
| T_c_to_r_C00249 (F) |
| T_c_to_r_C00024 (F) |
| R02057_r (R) |
| R01468_r (R) |
| R02038_r (R) |
| T_c_to_r_C00189 (R) |
| T_c_to_r_C00011 (R) |
| T_c_to_e_C00033 (R) |

**Table SIII B. Unique set of reactions impacted after integration of the bottleneck metabolites concentration ranges associated to the gene PF3D7_0111500.**

| Reaction ID[1] (Reaction directionalities[2]) |
|---|
| R00156_c (F) |
| R00570_c (F) |
| R04231_c (F) |
| R00155_c (B) |
| R00158_c (B) |
| R00512_c (B) |
| R00662_c (B) |

**Table SIII C. Unique set of reactions impacted after integration of the bottleneck metabolites concentration ranges associated to the gene PF3D7_0928900**

| Reaction ID[1] (Reaction directionalities[2]) |
|---|
| R00328_c (B) |
| R00332_c (B) |
| R00426_c (B) |
| R00428_c (B) |
| R00434_c (B) |
| R01234_c (B) |
| R01855_c (B) |
| R02090_c (B) |
| R04639_c (B) |
| R05046_c (B) |
| R05048_c (B) |

**Table SIII D. Common set of reactions impacted after integration of the bottleneck metabolites concentration ranges associated to the three genes PF3D7_1415700, PF3D7_0111500 and PF3D7_0928900.**

**Reaction ID[1] (Reaction directionalities[2])**

| | | | | | |
|---|---|---|---|---|---|
| **R00130_c (B)** | R02094_c (B) | R01195_a (B) | R07770_m (B) | T_c_to_e_C17023 (B) | T_c_to_e_C00137 (F) |
| **R00161_c (B)** | R02101_c (B) | R05633_a (B) | R07771_m (B) | T_c_to_e_C05198 (B) | T_c_to_e_C00183 (F) |
| **R00257_c (B)** | R02326_c (B) | R00570_a (B) | T_c_to_a_C00009 (B) | T_c_to_e_C00156 (B) | T_c_to_e_C00188 (F) |
| **R00416_c (B)** | R03361_c (B) | R00512_a (B) | T_c_to_a_C00013 (B) | T_c_to_e_C16240 (B) | T_c_to_e_C00712 (F) |
| **R00549_c (B)** | R03435_c (B) | R01015_a (B) | T_c_to_a_C00019 (B) | T_c_to_e_C00059 (B) | T_c_to_e_C01595 (F) |
| **R00619_c (B)** | R05692_c (B) | R05634_a (B) | T_c_to_a_C00073 (B) | T_c_to_e_C00255 (B) | T_c_to_e_C06424 (F) |
| **R00885_c (B)** | R08193_c (B) | R05636_a (B) | T_c_to_a_C00111 (B) | T_c_to_e_C00378 (B) | T_c_to_e_C00342 (F) |
| **R00888_c (B)** | R08639_c (B) | R05637_a (B) | T_c_to_a_C00129 (B) | T_c_to_e_C00407 (B) | R00174_c (R) |
| **R00965_c (B)** | R09247_c (B) | R05688_a (B) | T_c_to_a_C00235 (B) | T_c_to_e_C00187 (B) | R01021_c (R) |
| **R01658_c (B)** | R03469_c (B) | R08689_a (B) | T_c_to_a_C17023 (B) | T_c_to_e_C00120 (B) | R01799_c (R) |
| **R01665_c (B)** | R03005_c (B) | R04986_m (B) | T_c_to_a_C05198 (B) | T_c_to_e_C06453 (B) | R02030_c (R) |
| **R01724_c (B)** | R00104_c (B) | R04989_m (B) | T_c_to_a_C16240 (B) | T_c_to_e_C00725 (B) | R08969_c (R) |
| **R01802_c (B)** | R03035_c (B) | R04990_m (B) | T_c_to_m_C00725 (B) | T_c_to_r_C00114 (B) | R02240_c (R) |
| **R01870_c (B)** | R02018_c (B) | R05611_m (B) | T_c_to_m_C00013 (B) | R07390_c (F) | R01321_c (R) |
| **R01890_c (B)** | R01663_c (B) | R05615_m (B) | T_c_to_m_C00019 (B) | T_c_to_a_C00022 (F) | T_c_to_e_C00343 (R) |
| **R02003_c (B)** | R00289_c (B) | R06146_m (B) | T_c_to_m_C00021 (B) | T_c_to_e_C00041 (F) | |
| **R02017_c (B)** | R02058_c (B) | R00127_m (B) | T_c_to_m_C00156 (B) | T_c_to_e_C00078 (F) | |
| **R02019_c (B)** | R00291_c (B) | R04987_m (B) | T_c_to_m_C00129 (B) | T_c_to_e_C00114 (F) | |
| **R02024_c (B)** | R01818_c (B) | R04988_m (B) | T_c_to_m_C04216 (B) | T_c_to_e_C00123 (F) | |
| **R02093_c (B)** | R02061_c (B) | R05614_m (B) | T_c_to_m_C16240 (B) | T_c_to_e_C00135 (F) | |

[1]Reaction names as defined in iPfa. Metabolic reactions are defined with their R-5 digit identifier as obtained from the KEGG database. Transport reactions are marked with T_. Cellular compartment are defined with the reaction name: _r, endoplasmic reticulum; _c, cytosol. [2]Reaction directionalities obtained with Thermodynamics-based Flux Variability Analysis: (B) blocked, (F) forwards, (R) reverse. In this analysis no growth requirement was defined in iPfa. See the S4 Table for the reaction description.

**Studies on *in silico* minimal media (IMM).** The IMM analysis was performed following the strategy defined before [21]. Here, TFA was applied with the combined metabolomics data set integrated in iPfa (see the description of the thermodynamic curation of iPfa to know the experimental data that comprised this set). In this analysis, the growth rate was set to 0.06 $h^{-1}$ to study the nutritional requirements of normally growing *P. falciparum* (see the step on the definition of uptakes and secretions in the reconstruction process of iPfa to learn about the specific growth rate of *P. falciparum*). This means that the minimal nutritional requirements of iPfa were not underestimated (since a minimal growth was required) and were not overestimated (since growth was not allowed to reach extremely big and biologically irrelevant values).

The IMM analysis of iPfa suggested that iPfa requires as little as 23 substrates for growth and identified 10,032 alternative such IMM. There are 52 substrates that compose the alternative IMM and 16 of these substrates appear in all alternative IMM, here referred to as *constitutive substrates*. The remaining 36 substrates, here referred to as *non-constitutive substrates*, combine in groups of size seven to form the alternative IMM. In this way, each IMM, which is defined by 23 substrates, is composed of 16 constitutive substrates and seven non-constitutive substrates. *A priori* one would expect all the combinatorial possibilities of 36 substrates in groups of size seven to form the alternative IMM: 36!/((36-7)!*7!) = 8,347,680 combinations. However, the relatively low number of alternatives found in the IMM analysis suggests that not all combinations of the 36 substrates are possible, which indicates that not all substrates can substitute for each other.

**Identification of groups of substrates that can substitute for each other for growth.** Given the requirement of minimal utilization of substrates in the IMM studies, one would expect that some of the non-constitutive substrates that do not appear in the same IMM could substitute for each other. We created groups of non-constitutive substrates that did

not belong to the same IMM and found that 26 of the 36 components were substitutable, suggesting that each of these components serve a specific biosynthetic requirement. However, ten can be substituted by one or more than one substrate in the IMM, which indicates that they serve multiple biosynthetic requirements, as discussed for S-adenosyl-L-methionine in the main text. This result demonstrated that the non-appearance of two substrates in the same IMM is a necessary but not sufficient condition to define the substrate substitutability. We next looked at the molecular structure of the 36 non-constitutive substrates and identified the backbone moieties. Substrates were grouped if they never appeared in the same IMM and presented a common molecular substructure or backbone moiety.

To validate that the substrates with a backbone moiety were able to substitute for each other and support growth, we performed the following analysis: First, we tested that iPfa could not grow when all substrates in a group were removed from the *in silico* medium of 52 metabolites identified in the IMM. Second, we tested that under such conditions, the inclusion of each substrate of the group individually allowed simulated growth in iPfa. This analysis was also performed in the rich medium of 136 substrates to validate the groups of substrates that contained the three backbone moieties (the sources of carbon, phosphate and purine) indicated in Fig 3.

The 10,032 alternative IMM can be regenerated by combining all the constitutive metabolites with one non-constitutive metabolite from each group reported in Table 2. However, the following constraints should be considered:

- There should be at least one molecule that provides a ribose as source of carbon. In the IMM of iPfa, this ribose is provided by some molecules that also serve as source of pyridine ring or as source of DNA nucleotides (marked with * in Table 2).
- The combination between orthophosphate and S-Adenosylmethioninamine is not possible.

**Analysis of thermodynamic infeasibility in iPfa to utilize orthophosphate and S-Adenosylmethioninamine (SAM) as the only sources of phosphate and purines.**

The TFA of iPfa with metabolomics data integrated suggested that *P. falciparum* cannot utilize orthophosphate and SAM as the only sources of phosphate and purines, respectively.

We designed two sets of analyses to investigate the reason for this observation.

1. Identification of biomass building blocks (BBBs) whose production is impacted and limit the parasite's growth.
2. Identification of thermodynamic bottlenecks responsible for the thermodynamic infeasibility of *P. falciparum* to grow on orthophosphate and SAM.

In these analyses orthophosphate and SAM were forced to be the only sources of phosphate and purines. This was done by blocking the uptake of the remaining substrates that are sources of these backbone moieties in the *in silico* rich medium, which are defined in Figure 3. We performed TFA with the combined metabolomics data set.

*Analysis 1. Identification of biomass building blocks (BBBs) whose production is impacted and does not allow growth.*

When orthophosphate and SAM were forced to be the only sources of phosphate and purines no biomass growth was predicted. We tested the production of each BBB at a time to identify the cellular processes that could not be fueled with these metabolites. Interestingly, all BBBs could be individually produced. In this case, no growth was predicted because some BBBs could not be produced at the same time in the needed stoichiometric amounts. This result suggests that all cellular processes can function individually when orthophosphate and SAM are the only sources of phosphate and purine. However, the utilization of orthophosphate and SAM does not allow the required distribution of resources (e.g., carbon, energy or redox power) between the metabolic pathways needed for growth.

We next aimed to quantitatively understand the impact on the BBB production when orthophosphate and SAM were utilized. We compared the production of each BBB when all sources of phosphate and purine were available (as defined in Figure 3) and when only orthophosphate and SAM were used. The maximum production of 22 BBBs was considerably reduced in the latter scenario (Table SIV A). The most impacted BBB was ATP. This result suggests that *P. falciparum* might not be able to grow on orthophosphate and SAM because these precursors do not allow enough production of ATP for the cellular processes required for growth.

**Table SIV A. BBBs whose production is impacted when orthophosphate and SAM are the only sources of phosphate and purines.** The maximum production in such scenario (MPs) is compared with the maximum production of these BBBs when all sources of phosphate and purines are available (MPwt) in iPfa. The percentage of reduction in the production of each BBB is calculated as (MPwt - MPs) / MPwt * 100.

| Biomass building blocks impacted | | Max. Production, MPs (mmol/gDW/h) | Max. WT production, MPwt (mmol/gDW/h) | % Reduction in production |
|---|---|---|---|---|
| **C00002_c** | ATP | 16.7 | 100.0 | 83.3 |
| **C00013_c** | Diphosphate | 25.0 | 100.0 | 75.0 |
| **C00006_c** | NADP+ | 16.7 | 50.0 | 66.7 |
| **C00010_c** | CoA | 16.7 | 50.0 | 66.7 |
| **C00044_c** | GTP | 16.7 | 50.0 | 66.7 |
| **C00063_c** | CTP | 16.7 | 50.0 | 66.7 |
| **C00075_c** | UTP | 16.7 | 50.0 | 66.7 |
| **C00131_c** | dATP | 16.7 | 50.0 | 66.7 |
| **C00286_c** | dGTP | 16.7 | 50.0 | 66.7 |
| **C00458_c** | dCTP | 16.7 | 50.0 | 66.7 |
| **C00459_c** | dTTP | 16.7 | 50.0 | 66.7 |
| **C01245_c** | D-myo-Inositol 1,4,5-trisphosphate | 16.7 | 50.0 | 66.7 |
| **C00003_c** | NAD+ | 25.0 | 50.0 | 50.0 |
| **C00016_c** | FAD | 25.0 | 50.0 | 50.0 |
| **C00018_c** | Pyridoxal phosphate | 50.0 | 100.0 | 50.0 |
| **C00019_c** | S-Adenosyl-L-methionine | 50.0 | 100.0 | 50.0 |
| **C00043_c** | UDP-N-acetyl-D-glucosamine | 25.0 | 50.0 | 50.0 |
| **C00068_c** | Thiamin diphosphate | 25.0 | 50.0 | 50.0 |
| **C00096_c** | GDP-mannose | 25.0 | 50.0 | 50.0 |
| **C00325_c** | GDP-L-fucose | 25.0 | 50.0 | 50.0 |
| **C00029_c** | UDP-glucose | 25.0 | 50.0 | 50.0 |

| | | | | |
|---|---|---|---|---|
| **C00052_c** | UDP-D-galactose | 25.0 | 50.0 | 50.0 |
| **C05980_c** | Cardiolipin | 25.0 | 31.3 | 20.0 |
| **C16237_a** | Protein N6-(lipoyl)lysine | 0.2 | 0.2 | 6.6 |

We then investigated which BBBs could not be produced at the same time in the needed stoichiometric amounts. We developed a MILP formulation in which we aimed to minimize the number of BBBs whose production should be abolished to allow stoichiometric production of the remaining BBBs. The lower bound for the BBB demand reaction (LB) was set to 10% of the maximal production of each BBB (UB) at 0.06 $h^{-1}$:

$$\text{Minimize } \Sigma y_j$$
$$\text{s.t.}$$
$$\Sigma S_{ij} v_j = 0_i, \qquad i=1,\ldots M$$
$$v_{biomass}=0$$
$$LB_j \leq v_j \leq UB_j \qquad \forall j \in \text{Model}$$
$$LB_j (1-y_j) \leq v_j \leq UB_j (1-y_j) \qquad \forall j \in \text{Demand BBB reactions}$$
$$y_j = \{0,1\} \qquad \forall j \in \text{Demand BBB reactions}$$

We identified three such sets of BBBs (Table IV B).

**Table IV B. BBBs whose production should be abolished to allow stoichiometric production of the remaining BBBs.** All alternative solutions are reported.

| **Alternative sets** | **BBBs that imbalance production of biomass** | | | | | | |
|---|---|---|---|---|---|---|---|
| **Set 1** | ATP | | | | | | |
| **Set 2** | Sphingomyelin | | | | | | |
| **Set 3** | UDP-N-acetyl-D-glucosamine | UDP-D-galactose | UDP-glucose | CTP | UTP | dCTP | dTTP |

This result supports the previous observation of ATP limitation when *P. falciparum* grows on orthophosphate and SAM. And it further identifies the BBBs that are mostly impacted upon ATP limitation: the production of sphingomyelin, nucleotides and nucleotide sugars. The sets identified also indicate the parts of the metabolism that compete for the available resources such as ATP.

Furthermore, we identified that an unlimited production of Ubiquinone-8 in the mitochondrion or Sphingomyelin in the cytosol would allow growth of *P. falciparum* on orthophosphate and SAM. In this case, the production of ATP and the nucleotides is increased and there is stoichiometric production of the BBBs. This result suggests that *P. falciparum* might use the electron transport chain to increase the ATP production.

Note: We also defined the MILP formulation such that the minimal (LB) and maximal (UB) bounds of the BBB demands equal the maximum production rate of the BBBs at 0.06 $h^{-1}$. In this case, 24 additional sets appear (compared with the sets defined in Table IV B). ATP is a component of all these sets and it is combined with BBBs such as CoA, $FAD^+$, $NAD^+$, $NADP^+$, nucleotides, and nucleotide sugars. This result further supports the conclusion that there exits ATP limitation to produce biomass when *P. falciparum* grows on orthophosphate and SAM.

*Analysis 2. Identification of thermodynamic bottleneck that are responsible for the thermodynamic infeasibility to grow on orthophosphate and SAM.*

The infeasibility of iPfa to grow on orthophosphate and SAM was predicted with TFA and metabolomics data integrated. We applied the analysis of bottleneck metabolites to identify the metabolites responsible for the growth limitation.

Six bottleneck metabolites distributed in two sets of five bottleneck metabolites were identified (Table IV C).

**Table IV C. Bottleneck metabolites that render iPfa infeasible to grow on orthophosphate and SAM.** All alternative sets of bottleneck metabolites were identified as explained in the Materials and Methods section.

| Alternative sets | Bottleneck metabolites | | | | |
|---|---|---|---|---|---|
| **Set 1** | CTP[c] | CDP-choline[c] | L-Methionine[c] | ATP[c] | Choline[c] |
| **Set 2** | CTP[c] | CDP-choline[c] | L-Methionine[c] | ATP[c] | UTP[c] |

This result indicates that the concentrations of ATP together with other four metabolites render thermodynamic bottlenecks that do not allow growth on orthophosphate and SAM. The bottleneck metabolites identified participate in the production of phosphatidylcholine, sphingomyelin, nucleotides and nucleotide sugars. As suggested in the previous analysis (Table IV B), these metabolic processes might compete for the available resources. The results here presented further indicate that there might exist a thermodynamic coupling between the production of sphingomyelin, nucleotides and nucleotide sugars through the concentrations of the six bottleneck metabolites.

We next investigated where the thermodynamic bottlenecks were located in the metabolism of *P. falciparum*. We identified the reactions whose directionality was impacted upon integration of the bottleneck metabolite concentrations. We compared the directionality of the reactions in iPfa in two conditions: when the generic concentration ranges (1 μM - 50 mM) were defined for all intracellular metabolites, and when the experimental concentration ranges of the bottleneck metabolites in each set was further integrated. The directionality of the reactions was identified through Thermodynamic Variability Analysis (TVA) [4] as explained in the Materials and Methods section. Both sets of bottleneck metabolites impacted the same reaction directionalities. In these analyses all sources of phosphate and purine were available (as defined in Figure 3).

We identified two reactions whose directionality changed upon integration of the bottleneck metabolite concentrations (Table IV D). The reactions impacted participate in the production of phosphatidylcholine and imply the consumption of ATP.

**Table IV D. Reactions in iPfa whose directionality is impacted after integration of the bottleneck metabolites concentration ranges.** The full description of the reaction (as defined in iPfa) is also provided.

| Reaction ID[1] (directionality[2]) | Enzyme | E. C. | Reaction description | Gene associated |
|---|---|---|---|---|
| **R01021_c (F)** | ATP:choline phosphotransferase | 2.7.1.32 | ATP[c] + Choline[c] <=> ADP[c] + Choline phosphate[c] | (PF3D7_1401800) |
| **T_c_to_e_C00114 (R)** | NA | NA | Choline[c] <=> Choline[e] | NA |

[1]Reaction names as defined in iPfa. Metabolic reactions are defined with their R-5 digit identifier as obtained from the KEGG database. Transport reactions are marked with T_. Cellular compartment are defined with the reaction name: _r, endoplasmic reticulum; _c, cytosol. [2]Reaction directionalities obtained with Thermodynamics-based Flux Variability Analysis: (B) blocked, (F) forwards, (R) reverse.

**Gene essentiality in the IMM.** The results on essential genes that can become essential upon substrate inaccessibility reported in S5 Table were obtained by defining one-by-one the 10,032 alternative *in silico* minimal media (IMM), which are composed uniquely of 23 substrates. The composition of all IMM can be derived from the Table 2. The methods used for the identification of the essential genes and reactions in these analyses are reported in S2, S3 and S5 Tables. The studies on essentiality with TFA follow the same principles as with FBA and further account for thermodynamic constraints, which reduce the feasible solution space from FBA by allowing only thermodynamically feasible solution of fluxes (see the studies on Reduction of Uncertainty). The constraints in the reaction directionalities are responsible for the identification of the additional essential genes observed with TFA. At the same time, this implies that the genes identified as essential with FBA are always a subset of the genes identified as essential with TFA.

Although we expect that most of the genes that TFA predicted to be essential in the *in silico* rich medium (S2 Table) will also be essential *in vivo*, some of the genes identified as non-essential in this study could be essential under certain *in vivo* conditions. The model and the IMM analysis framework presented here can be used to further explore which substrates are not accessible by the cell or are not available in sufficient quantities to *P. falciparum* inside the host. For example, a future comparison of *in vivo* knockout high-throughput data and this essentiality analysis on the IMM will advance our understanding of the substrates that are accessible to the malaria parasite in the host.

**Substrate channeling integration within TFA.** Substrate channeling between two enzymes defines the process in which the intermediate produced by one enzyme is transferred to the next enzyme without complete mixing with the bulk phase [57]. This process might occur to facilitate the flux through a metabolic pathway.
The presence of substrate channeling between two enzymes ($E_1$ and $E_2$) was simulated within the TFA framework. The reactions $R_1$ and $R_2$ catalyzed by $E_1$ and $E_2$ were lumped to define an overall reaction ($L= R_1+R_2$). The overall reaction eliminates the common intermediates and recycled metabolites, since they are produced in the first reaction $R_1$ and consumed in the second reaction $R_2$ and vice versa, respectively. For example, if $R_1$ involves the transformation A + B → I + C and $R_2$ is defined as I + D → P + B, the final lumped or overall reaction L is defined as A + D → P + C. The overall reaction L does not involve the intermediate metabolite I and the recycled metabolite B. It is important to note that reactions $R_1$ and $R_2$ should be first defined in the direction that satisfies mass balances and allows flux through the pathway of study. Then the lumped reaction can be formed. For example, if the pathway of study contains reactions $R_1$ and $R_2$ and normally the starting metabolite is A and the product of the pathway is P, the reactions $R_1$ and $R_2$ should be defined as A + B → I + C and I + D → P + B, respectively, and not as A + B → I + C and P + B → I + D. In this study, the reactions of the pyrimidine biosynthesis pathway and the Kennedy pathway were defined to allow production of UMP and PE, respectively. The overall reaction was then allowed to be bidirectional. Thermodynamic properties were calculated for the overall reactions and TFA and Thermodynamic Variability Analysis

(TVA) [4] were performed to determine the thermodynamically feasible directionality of the overall reactions. Substrate channeling was suggested when the directionality of the overall reaction allowed flux through the biosynthetic pathway that would be otherwise thermodynamically infeasible. The directionality of the reactions was known from the sign of the range in the $\Delta_r G'$ obtained from TVA.

We hypothesize that substrate channeling might exist in three pathways (two of which are discussed in the main text):

- The Kennedy pathway: we defined an overall reaction between ethanolamine phosphate and PE through the enzymes E.C. 2.7.7.14 and E.C. 2.7.8.1 (R02038_r and R02057_r).
- The pyrimidine biosynthesis pathway: we defined an overall reaction between carbamoyl phosphate to (S)-dihydroorotate through the enzymes E.C. 2.1.3.2 and E.C. 3.5.2.3 (R01397_c and R01993_c).
- The multi enzymatic step through S-Adenosyl-L-methionine:(methyl)ethanolamine-phosphate N-methyltransferase: we defined an overall reaction between ethanolamine phosphate and choline phosphate through the enzyme E.C. 2.1.1.103 (R02037_c, R06868_c, and R06869_c).

It is important to note, that the presence of a channel in the Kennedy pathway, does not allow the correct identification of the gene *PF3D7_0927900* as essential in iPfa using TFA with metabolomics data integrated. Instead, this gene appears in six additional synthetic lethal pairs (Table SV).

**Table SV. Additional synthetic lethal pairs identified when channeling in the Kennedy pathway is simulated.** All pairs involve the gene *PF3D7_0927900.*

| Pairs of essential genes in iPfa | | Metabolic tasks impacted upon knockout of each pair | |
|---|---|---|---|
| PF3D7_1216200 | PF3D7_0927900 | C00350_r | Phosphatidylethanolamine |
| PF3D7_1419800.1 | PF3D7_0927900 | C00350_r | Phosphatidylethanolamine |
| PF3D7_0923800.2 | PF3D7_0927900 | C00350_r | Phosphatidylethanolamine |
| PF3D7_1124600 | PF3D7_0927900 | C00350_r | Phosphatidylethanolamine |
| PF3D7_1347700 | PF3D7_0927900 | C00350_r | Phosphatidylethanolamine |
| PF3D7_0628300 | PF3D7_0927900 | C00350_r | Phosphatidylethanolamine |

The gene *PF3D7_0927900* encodes a phosphatidyl serine carboxylase (E.C. 4.1.1.65) and has been observed as essential in the blood stages of *P. falciparum* using drugs [58]. We need to simulate different scenarios to identify *PF3D7_0927900* as essential while channeling is present in the Kennedy pathway. For example, we can consider that there is a maximal allowable flux through E.C. 4.1.1.65 and the Kennedy pathway and therefore both pathways are required to maintain optimal growth.
In addition, regulation might exist between these redundant pathways.

In the same way, the presence of a channel in the pyrimidine biosynthetic pathway implies that the backbone moiety in orotate and (S)-dihydroorotate can be synthesized by the cell. The uptake of this backbone moiety is then non-essential. Experimental studies have suggested that orotate uptake of *in vitro* extracellularly developing malaria parasites are a direct measurement of parasites growth [59]. The presence of orotate in the medium has been shown to inhibit the function of the DHOase enzyme [60]. This observation can be reflected in the model if we define a regulatory loop between orotate uptake and the pyrimidine biosynthesis pathway.

**Using iPfa to study its metabolism in the blood and liver stages.** iPfa can be implemented to study the metabolism of *P. falciparum* during the blood and liver stages. Here, we suggest some modifications of iPfa that would render better context-specific predictions.

For modeling of blood-stage specific metabolism, the protein N6-(lipoyl)lysine in apicoplast (whose metabolite ID in iPfa is C16237_a) should be removed from the biomass. This metabolite is a precursor of the lipoyl-ACP, which is a cofactor of the pyruvate dehydrogenase complex in apicoplast, and its production requires the fatty acid synthesis II (FAS II) pathway in this compartment to be active. This pathway has been experimentally observed as dispensable during the blood stages but essential during the liver stages [61] (S2 Table).

For modeling the liver-stage specific metabolism, the uptake of hemoglobin (whose rxn ID in iPfa is T_c_to_e_C05781) should be prohibited and the reaction that represents the hemoglobin digestion (HBDG_c) should be blocked or erased, since this molecule is not supposed to be accessible inside the hepatocyte cell.

**Part 3. Comparison between iPfa and the previous models of *P. falciparum* metabolism iTH366 [62] and PlasmoNet [63].**

The model iPfa was compared at its initial and final reconstruction stages with previous models of *P. falciparum* metabolism. Here we provide the final comparison of the metabolic reactions in terms of E.C. identifiers (Fig SIII A), the BBBs (Fig SIII B) and the genes included (Fig SIII C).

The comparison of metabolic reactions shows that the models share a big set of enzymatic functions, i.e. 230 ECs. The model PlasmoNet involves the highest number of metabolic reactions, i.e. 475 ECs, followed by iPfa and iTH366. Since PlasmoNet does not include genes, it was not possible to perform a critical evaluation of the missing ECs in iPfa. If the unique metabolic reactions to PlasmoNet were not orphan, we would have considered their integration in iPfa. The unique metabolic reactions to iTH366 were not included in iPfa because they were orphan reactions or they were associated to genes with uncertain annotation.

In terms of BBBs, iPfa presents the highest description of metabolic tasks, followed by PlasmoNet and iTH366. The metabolic tasks in iTH366 that are not included in iPfa involve the production of ferric ions ($Fe^{2+}$ and $Fe^{3+}$) and menaquinone. PlasmoNet further requires the production of (9Z)-hexadecenoic acid, 3'',5''-Cyclic AMP, active-glutathione-transferase, active-glyoxalase-II, active-SAH-hydrolase, active-thioredoxin-reductase, phosphatidate, protein C-terminal S-farnesyl-L-cysteine methyl ester, Fe-S-protein, glycoprotein with the oligosaccharide chain attached by N-glycosyllinkage to protein L-asparagine, H+-pumped, hemozoin, and protein N6-[(R)-4-amino-2-hydroxybutyl]-L-lysine. The metabolic tasks that are unique to iPfa are tetradecanoic acid, biotin, 5-methyltetrahydrofolate, methylcobalamin, geranylgeranyl diphosphate, UDP-N-acetyl-D-glucosamine, GDP-L-fucose, UDP-glucose, and UDP-D-galactose.

The genes in iPfa could only be compared with iTH366, since no genes are provided with PlasmoNet. The models iPfa and iTH366 share a big set of 245 genes. The previous model iTH366 includes 122 genes that were carefully evaluated at the initial stages of the reconstruction of iPfa (July-December 2014). These genes were not included in iPfa

because they were associated to putative annotations in PlasmoDB and no reference in the literature was found to support the suggested annotation.

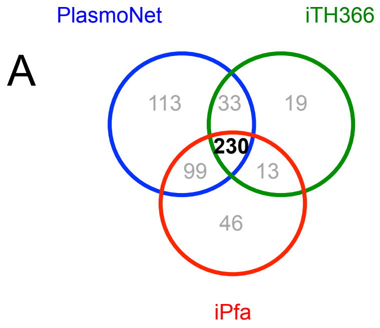


**Fig SIII A.** Comparison of metabolic reactions (in terms of E.C. identifiers)

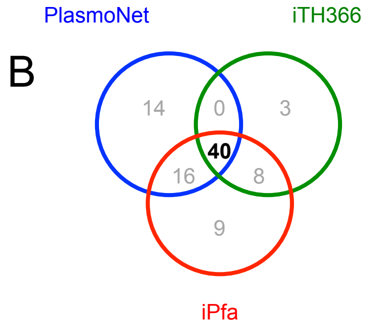


**Fig SIII B.** Comparison of biomass building blocks

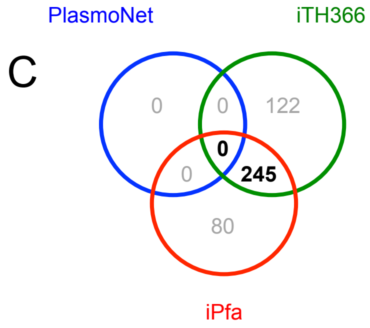


**Fig SIII C.** Comparison of genes

We further compared iPfa with the previous models in terms of ad hoc reaction directionalities, blocked reactions and essential genes (Table SVI). The models iPfa and iTH366 share similar characteristics in terms of blocked reactions and essential genes. This occurs although iPfa is much more relaxed in terms of substrate availability, metabolite transportability and *ad hoc* reaction directionalities. To this end, iPfa represents an ideal scaffold for the integration of context specific information and the identification of an increased number of essential genes for specific life stages.

**Table SVI. Functional comparison of iPfa with the previous models of *P. falciparum*, PlasmoNet and iTH366.**

| | PlasmoNet | iTH366 | iPfa |
|---|---|---|---|
| **No. *ad hoc* reaction directionalities (%)** | 0 (0%) | 590 (59%) | 185 (14%) |
| **No. Blocked reactions (%)** | NA | 334 (33%) | 313 (24%) |
| **No. Essential genes (%)** | NA | 57 (16%) | 55 (17%) |

## Part 4. Further information on the materials and methods used in the reconstruction and analyses of iPfa

**Software and databases.** We solved FBA with the COBRA Toolbox v2.0 [64] and TFA using MATLAB (R2014a and R2014b) and CPLEX (ILOG IBM v12.51) as the linear solver. For the gap-filling algorithm we specified a CPLEX limit time of 500 seconds. We reconstructed and curated the metabolic network of iPfa using the RAVEN Toolbox v1.08 [1] with mosek v7 in MATLAB (R2014a and R2014b). We obtained the reactions and metabolites present in iPfa from the KEGG database (between July 2014 and December 2015). Marvin was used for drawing, displaying and characterizing the chemical substructures, Marvin Beans (16.7.25.0), 2016, ChemAxon (http://www.chemaxon.com) with molfiles from the KEGG database [11].

## References SI

1. Agren R, Liu L, Shoaie S, Vongsangnak W, Nookaew I, Nielsen J. The RAVEN toolbox and its use for generating a genome-scale metabolic model for *Penicillium chrysogenum*. PLoS Comput Biol. 2013;9(3):e1002980.

2. Desai SA. Insights gained from *P. falciparum* cultivation in modified media. ScientificWorldJournal. 2013;2013:363505.

3. Krebs HA. Chemical composition of blood plasma and serum. Annu Rev Biochem. 1950 (19):409-30. PubMed PMID: 14771836.

4. Henry CS, Broadbelt LJ, Hatzimanikatis V. Thermodynamics-based metabolic flux analysis. Biophys J. 2007;92(5):1792-805.

5. Jankowski MD, Henry CS, Broadbelt LJ, Hatzimanikatis V. Group contribution method for thermodynamic analysis of complex metabolic networks. Biophys J. 2008;95(3):1487-99. PubMed PMID: 18645197. Pubmed Central PMCID: PMC2479599.

6. Jol SJ, Kümmel A, Hatzimanikatis V, Beard Da, Heinemann M. Thermodynamic calculations for biochemical transport and reaction processes in metabolic networks. Biophys J. 2010;99(10):3139-44. PubMed PMID: 21081060. Pubmed Central PMCID: PMC2980713.

7. Jensen MD, Conley M, Helstowski LD. Culture of *Plasmodium falciparum*: the role of pH, glucose, and lactate. J Parasitol. 1983;69(6):1060-7.

8. Martin RE, Kirk K. Transport of the essential nutrient isoleucine in human erythrocytes infected with the malaria parasite *Plasmodium falciparum*. Blood. 2007;109(5):2217-24.

9. Aurrecoechea C, Brestelli J, Brunk BP, Dommer J, Fischer S, Gajria B, et al. PlasmoDB: a functional genomic database for malaria parasites. Nucleic Acids Res. 2009 Jan;37(Database issue):D539-43. PubMed PMID: 18957442. Pubmed Central PMCID: 2686598.

10. Gardner MJ, Hall N, Fung E, White O, Berriman M, Hyman RW, et al. Genome sequence of the human malaria parasite *Plasmodium falciparum*. Nature. 2002 Oct 3;419(6906):498-511. PubMed PMID: 12368864. Pubmed Central PMCID: 3836256.

11. Kanehisa M, Goto S. KEGG : Kyoto Encyclopedia of Genes and Genomes. Nucleic Acids Res. 2000;28(1):27-30.

12. Skelton FS, Lunan KD, Folkers K, Schnell JV, Siddiqui WA, Geiman QM. Biosynthesis of ubiquinones by malarial parasites. I. Isolation of [14C]ubiquinones from cultures of rhesus monkey blood infected with *Plasmodium knowlesi*. Biochemistry. 1969 Mar;8(3):1284-7. PubMed PMID: 4976406.

13. Oppenheim RD, Creek DJ, Macrae JI, Modrzynska KK, Pino P, Limenitakis J, et al. BCKDH: the missing link in apicomplexan mitochondrial metabolism is required for full virulence of *Toxoplasma gondii* and *Plasmodium berghei*. PLoS Pathog. 2014;10(7):e1004263.

14. ApiLoc - A database of published protein sub-cellular localization in Apicomplexa. 2011 [cited 2014-2015]. Available from: http://apiloc.biochem.unimelb.edu.au/apiloc/apiloc.

15. Ginsburg H, Tilley L. *Plasmodium falciparum* metabolic pathways (MPMP) project upgraded with a database of subcellular locations of gene products. Trends Parasitol. 2011;27(7):285-6.

16. Roos DS, Crawford MJ, Donald RG, Kissinger JC, Klimczak LJ, Striepen B. Origin, targeting, and function of the apicomplexan plastid. Curr Opin Microbiol. 1999 Aug;2(4):426-32. PubMed PMID: 10458993.

17. Emanuelsson O, Brunak S, von Heijne G, Nielsen H. Locating proteins in the cell using TargetP, SignalP and related tools. Nat Protoc. 2007;2(4):953-71. PubMed PMID: 17446895.

18. Claros MG. MitoProt, a Macintosh application for studying mitochondrial proteins. Comput Appl Biosci. 1995 Aug;11(4):441-7. PubMed PMID: 8521054.
19. Cilingir G, Broschat SL, Lau AO. ApicoAP: the first computational model for identifying apicoplast-targeted proteins in multiple species of Apicomplexa. PLoS One. 2012;7(5):e36598. PubMed PMID: 22574192. Pubmed Central PMCID: 3344922.
20. Kirk K, Lehane AM. Membrane transport in the malaria parasite and its host erythrocyte. Biochem J. 2014 Jan 1;457(1):1-18. PubMed PMID: 24325549.
21. Tymoshenko S, Oppenheim RD, Agren R, Nielsen J, Soldati-Favre D, Hatzimanikatis V. Metabolic Needs and Capabilities of *Toxoplasma gondii* through Combined Computational and Experimental Analysis. PLoS Comput Biol. 2015;11(5):1-28.
22. Mullin KA, Lim L, Ralph SA, Spurck TP, Handman E, McFadden GI. Membrane transporters in the relict plastid of malaria parasites. Proc Natl Acad Sci USA. 2006 Jun 20;103(25):9572-7. PubMed PMID: 16760253. Pubmed Central PMCID: 1480448.
23. Forth T. Metabolic Systems Biology of the Malaria Parasite. UK: The University of Leeds; 2012.
24. Palacpac NM, Hiramine Y, Mi-ichi F, Torii M, Kita K, Hiramatsu R, et al. Developmental-stage-specific triacylglycerol biosynthesis, degradation and trafficking as lipid bodies in *Plasmodium falciparum*-infected erythrocytes. J Cell Sci. 2004 Mar 15;117(Pt 8):1469-80. PubMed PMID: 15020675.
25. Hsiao LL, Howard RJ, Aikawa M, Taraschi TF. Modification of host cell membrane lipid composition by the intra-erythrocytic human malaria parasite *Plasmodium falciparum*. Biochem J. 1991 Feb 15;274(Pt 1):121-32. PubMed PMID: 2001227. Pubmed Central PMCID: 1149929.
26. Mi-Ichi F, Kita K, Mitamura T. Intraerythrocytic *Plasmodium falciparum* utilize a broad range of serum-derived fatty acids with limited modification for their growth. Parasitology. 2006 Oct;133(Pt 4):399-410. PubMed PMID: 16780611.
27. Neidhardt FC, Ingraham J, Schaechter M. Physiology of the Bacterial Cell: A Molecular Approach. Sunderland, Massachusetts: Sinauer Associates; 1990.
28. Thiele I, Palsson BO. A protocol for generating a high-quality genome-scale metabolic reconstruction. Nat Protoc. 2010;5(1):93-121.
29. Pfaller MA, Krogstad DJ, Parquette AR, Nguyen-Dinh P. *Plasmodium falciparum*: stage-specific lactate production in synchronized cultures. Exp Parasitol. 1982 Dec;54(3):391-6. PubMed PMID: 6759150.
30. Ginsburg H. Abundant proton pumping in *Plasmodium falciparum*, but why? Trends Parasitol. 2002 Nov;18(11):483-6. PubMed PMID: 12473363.
31. Mysliwski A, Lass P. Increase of size and dry mass of mouse erythrocytes depending on age of donors. Mech Ageing Dev. 1985 Feb;29(2):107-10. PubMed PMID: 3974304.
32. Liu J, Gluzman IY, Drew ME, Goldberg DE. The role of *Plasmodium falciparum* food vacuole plasmepsins. J Biol Chem. 2005 Jan 14;280(2):1432-7. PubMed PMID: 15513918.
33. Gazzinelli RT, Kalantari P, Fitzgerald KA, Golenbock DT. Innate sensing of malaria parasites. Nat Rev Immunol. 2014 Nov;14(11):744-57. PubMed PMID: 25324127.
34. Soh KC, Hatzimanikatis V. Network thermodynamics in the post-genomic era. Curr Opin Microbiol. 2010;13(3):350-7.
35. Soh KC, Hatzimanikatis V. Constraining the flux space using thermodynamics and integration of metabolomics data. In: Krömer JO, Nielsen LK, Blank LM, editors. Metabolic Flux Analysis. 1191. New York: Springer Protocols; 2014. p. 49-63.
36. Ataman M, Hatzimanikatis V. Heading in the right direction: thermodynamics-based network analysis and pathway engineering. Curr Opin Biotechnol. 2015;36:176-82.

37. Teng R, Junankar PR, Bubb Wa, Rae C, Mercier P, Kirk K. Metabolite profiling of the intraerythrocytic malaria parasite *Plasmodium falciparum* by (1)H NMR spectroscopy. NMR Biomed. 2009;22(3):292-302.
38. Teng R, Lehane Adele M, Winterberg M, Shafik Sarah H, Summers Robert L, Martin Rowena E, et al. 1H-NMR metabolite profiles of different strains of *Plasmodium falciparum*. Biosci Rep. 2014;34(6):685-99.
39. Vo Duy S, Besteiro S, Berry L, Perigaud C, Bressolle F, Vial HJ, et al. A quantitative liquid chromatography tandem mass spectrometry method for metabolomic analysis of *Plasmodium falciparum* lipid related metabolites. Anal Chim Acta. 2012;739:47-55.
40. Sanz S, Bandini G, Ospina D, Bernabeu M, Mariño K, Fernández-Becerra C, et al. Biosynthesis of GDP-fucose and other sugar nucleotides in the blood stages of *Plasmodium falciparum*. J Biol Chem. 2013;288(23):16506-17.
41. Saliba KJ, Kirk K. pH regulation in the intracellular malaria parasite, *Plasmodium falciparum*. H(+) extrusion via a V-type H(+)-ATPase. J Biol Chem. 1999 Nov 19;274(47):33213-9. PubMed PMID: 10559194.
42. Allen RJ, Kirk K. The membrane potential of the intraerythrocytic malaria parasite *Plasmodium falciparum*. J Biol Chem. 2004 Mar 19;279(12):11264-72. PubMed PMID: 14630911.
43. Orij R, Postmus J, Ter Beek A, Brul S, Smits GJ. In vivo measurement of cytosolic and mitochondrial pH using a pH-sensitive GFP derivative in *Saccharomyces cerevisiae* reveals a relation between intracellular pH and growth. Microbiology. 2009 Jan;155(Pt 1):268-78. PubMed PMID: 19118367. Epub 2009/01/02. eng.
44. Vercesi AE, Bernardes CF, Hoffmann ME, Gadelha FR, Docampo R. Digitonin permeabilization does not affect mitochondrial function and allows the determination of the mitochondrial membrane potential of *Trypanosoma cruzi* in situ. J Biol Chem. 1991 Aug 5;266(22):14431-4. PubMed PMID: 1860850.
45. Mikkelsen RB, Wallach DF, Van Doren E, Nillni EA. Membrane potential of erythrocytic stages of *Plasmodium chabaudi* free of the host cell membrane. Mol Biochem Parasitol. 1986 Oct;21(1):83-92. PubMed PMID: 3773936.
46. Uyemura SA, Luo S, Moreno SN, Docampo R. Oxidative phosphorylation, Ca(2+) transport, and fatty acid-induced uncoupling in malaria parasites mitochondria. J Biol Chem. 2000 Mar 31;275(13):9709-15. PubMed PMID: 10734123.
47. Uyemura SA, Luo S, Vieira M, Moreno SN, Docampo R. Oxidative phosphorylation and rotenone-insensitive malate- and NADH-quinone oxidoreductases in *Plasmodium yoelii yoelii* mitochondria in situ. J Biol Chem. 2004 Jan 2;279(1):385-93. PubMed PMID: 14561763.
48. Vercesi AE, Rodrigues CO, Uyemura SA, Zhong L, Moreno SN. Respiration and oxidative phosphorylation in the apicomplexan parasite *Toxoplasma gondii*. J Biol Chem. 1998 Nov 20;273(47):31040-7. PubMed PMID: 9813002.
49. Ludovico P, Sansonetty F, Corte-Real M. Assessment of mitochondrial membrane potential in yeast cell populations by flow cytometry. Microbiology. 2001 Dec;147(Pt 12):3335-43. PubMed PMID: 11739765. Epub 2001/12/12. eng.
50. Henry CS, Jankowski MD, Broadbelt LJ, Hatzimanikatis V. Genome-scale thermodynamic analysis of *Escherichia coli* metabolism. Biophys J. 2006;90(4):1453-61.
51. Schomburg I, Chang A, Ebeling C, Gremse M, Heldt C, Huhn G, et al. BRENDA, the enzyme database: updates and major new developments. Nucleic Acids Res. 2004 Jan 1;32(Database issue):D431-3. PubMed PMID: 14681450. Pubmed Central PMCID: 308815. Epub 2003/12/19. eng.

52. Orth JD, Palsson BO. Systematizing the generation of missing metabolic knowledge. Biotechnol Bioeng. 2010 Oct 15;107(3):403-12. PubMed PMID: 20589842. Epub 2010/07/01. eng.
53. Orth JD, Thiele I, Palsson BO. What is flux balance analysis? Nat Biotechnol. 2010;28(3):245-8.
54. Atkinson DE. The energy charge of the adenylate pool as a regulatory parameter. Interaction with feedback modifiers. Biochemistry. 1968 Nov;7(11):4030-4. PubMed PMID: 4972613. Epub 1968/11/01. eng.
55. Mahadevan R, Schilling CH. The effects of alternate optimal solutions in constraint-based genome-scale metabolic models. Metab Eng. 2003 Oct;5(4):264-76. PubMed PMID: 14642354.
56. Kiparissides A, Hatzimanikatis V. Thermodynamics-based Metabolite Sensitivity Analysis in metabolic networks. Metab Eng. 2016 Nov 12. PubMed PMID: 27845184.
57. Spivey HO, Ovadi J. Substrate channeling. Methods. 1999 Oct;19(2):306-21. PubMed PMID: 10527733.
58. Roggero R, Zufferey R, Minca M, Richier E, Calas M, Vial H, et al. Unraveling the mode of action of the antimalarial choline analog G25 in *Plasmodium falciparum* and *Saccharomyces cerevisiae*. Antimicrob Agents Chemother. 2004 Aug;48(8):2816-24. PubMed PMID: WOS:000222998300005. English.
59. Trager W. Malaria parasites (Plasmodium lophurae) developing extracellularly in vitro: incorporation of labeled precursors. J Protozool. 1971 Aug;18(3):392-9. PubMed PMID: 5132315.
60. Krungkrai SR, Wutipraditkul N, Krungkrai J. Dihydroorotase of human malarial parasite *Plasmodium falciparum* differs from host enzyme. Biochem Biophys Res Commun. 2008 Feb 15;366(3):821-6. PubMed PMID: 18082626.
61. Shears MJ, Botte CY, McFadden GI. Fatty acid metabolism in the *Plasmodium* apicoplast: Drugs, doubts and knockouts. Mol Biochem Parasitol. 2015 Jan-Feb;199(1-2):34-50. PubMed PMID: 25841762.
62. Plata G, Hsiao T-L, Olszewski KL, Llinás M, Vitkup D. Reconstruction and flux-balance analysis of the *Plasmodium falciparum* metabolic network. Mol Syst Biol. 2010;6(408).
63. Huthmacher C, Hoppe A, Bulik S, Holzhütter H-G. Antimalarial drug targets in *Plasmodium falciparum* predicted by stage-specific metabolic network analysis. BMC Syst Biol. 2010;4(120).
64. Schellenberger J, Que R, Fleming RM, Thiele I, Orth JD, Feist AM, et al. Quantitative prediction of cellular metabolism with constraint-based models: the COBRA Toolbox v2.0. Nat Protoc. 2011 Sep;6(9):1290-307. PubMed PMID: 21886097. Pubmed Central PMCID: 3319681.
